# A chromatin-associated pool of Aurora A controls kinetochore-microtubule attachments to ensure chromosome biorientation

**DOI:** 10.1126/sciadv.aed5283

**Published:** 2026-05-06

**Authors:** Johnathan L. Meaders, Alyssa A. Rodriguez, Smriti Variyar, SungWoo Park, Alessandro E. Cirulli, Karen Oegema, Kevin D. Corbett, Arshad Desai

**Affiliations:** ^1^ Department of Cell and Developmental Biology, School of Biological Sciences, University of California San Diego, La Jolla, CA 92093, USA.; ^2^ Department of Cellular and Molecular Medicine, University of California San Diego, La Jolla, CA 92093, USA.; ^3^ Department of Molecular Biology, School of Biological Sciences, University of California San Diego, La Jolla, CA 92093, USA.

## Abstract

Accurate chromosome segregation requires dynamic kinetochore-microtubule attachments that, under the regulation of Aurora family kinases, biorient and align replicated chromosomes. In *Caenorhabditis elegans*, Aurora A acts with the TPX2-related activator TPXL-1 to regulate these attachments and control spindle length. We show that, in addition to prominent spindle pole localization, TPXL-1–AurA has a chromatin-associated pool positioned between the sister kinetochores. Structural modeling and biochemical analysis support TPXL-1 directly recognizing the nucleosome acidic patch via an arginine anchor. Disrupting this interaction selectively removed chromatin-bound TPXL-1–AurA and caused chromosome missegregation, whereas elevation of the chromatin pool disrupted chromosome alignment. These opposing perturbations inversely affected kinetochore recruitment of the microtubule-binding Ska complex. These results support spatially distinct TPXL-1–AurA populations acting sequentially, with the spindle pole pool controlling spindle length by switching kinetochores out of a depolymerization-coupled state, and the chromatin pool controlling attachment stabilization to ensure biorientation prior to anaphase.

## Teaser

Spatially distinct pools of Aurora A act on kinetochores to ensure chromosome biorientation and control spindle length.

## INTRODUCTION

Faithful chromosome segregation during cell division is essential for maintaining genomic stability and preventing aneuploidy, a hallmark of cancer and many developmental disorders. During mitosis, replicated sister chromatids are separated by the microtubule-based mitotic spindle, which exerts pulling forces to move the sisters toward opposite poles. Chromosomes assemble kinetochores—large, multiprotein structures that connect centromeric chromatin to spindle microtubules (*1*, *2*). Kinetochore-spindle microtubule attachments must be dynamic yet tightly regulated to allow continual error correction until all chromosomes achieve biorientation, the configuration in which each chromatid is connected exclusively to microtubules from one spindle pole and sister chromatids are connected to opposite spindle poles. The coordinated dynamics of kinetochore-microtubule attachments are also central to spindle length regulation and for bioriented chromosomes to align at the spindle midplane to form a metaphase plate. Together, these mechanisms ensure that when cohesion between sister chromatids is dissolved at anaphase onset, the forces transmitted through kinetochore microtubules faithfully segregate one copy of the replicated genome to each daughter cell.

Regulation of kinetochore-microtubule attachments to ensure chromosome biorientation is complex and involves multiple activities, the most prominent of which is phosphorylation by Aurora family kinases—Aurora A (AurA) and Aurora B (AurB) (*3*). Studies in budding yeast, which has a single Aurora kinase, followed by work in metazoans, highlighted a central role for AurB, acting in the context of the chromosomal passenger complex that localizes between the sister kinetochores, in ensuring chromosome biorientation (*4*–*6*). In metazoans, AurA also plays a key role in kinetochore regulation (*7*–*14*). AurA exists in multiple pools bound to distinct regulatory factors—including TPX2, CEP192, and BORA in humans—and localizes prominently to spindle poles, mediated by CEP192 and TPX2 (*15*). In addition, AurA has been observed on mitotic chromatin adjacent to kinetochore-microtubule attachments in mammals and in *Caenorhabditis elegans* (*7*, *10*, *11*, *16*), but the function of this chromatin-associated population has remained unclear due to the absence of tools that can selectively perturb it.

Here, we use the *C. elegans* embryo to study the functions of AurA in kinetochore regulation. In *C. elegans*, AurA activated by the TPX2-like cofactor TPXL-1 (TPXL-1–AurA) regulates kinetochore-microtubule attachments (*8*, *12*, *17*), whereas AurB plays a minor role (*8*). Depleting TPXL-1 or mutating it to prevent AurA binding traps kinetochores in a persistent depolymerization-coupled state, causing spindle poles to be pulled inward after nuclear envelope breakdown (NEBD), resulting in a rapid reduction in spindle length (“spindle collapse”); this collapse can be suppressed by preventing outer kinetochore assembly (*8*, *12*). These results indicate that TPXL-1–AurA is required for kinetochores to exit from a persistent depolymerization-coupled state upon NEBD. However, it has remained unclear whether this regulation is mediated by the spindle pole or chromatin-associated pools of AurA, and whether promoting the transition out of the depolymerization-coupled state represents the only function of AurA at kinetochores.

In this study, we define the mechanism by which AurA is recruited to chromatin as well as identify a means to elevate it at this cellular location. By selectively removing or elevating the chromatin-associated pool of AurA, we show that this pool is dispensable for the transition of kinetochores out of the depolymerization-coupled state, suggesting that the spindle pole–associated population is sufficient for this function, which is critical for regulation of spindle length. However, we find that the chromatin-associated population is important to ensure biorientation of all chromosomes prior to anaphase onset. We show that the chromatin-associated AurA pool regulates SKA complex recruitment to kinetochores, potentially through phosphorylation of the Ndc80 N-terminal tail, and that its precise level on chromatin is important to biorient and align chromosomes. Together, our findings reveal how spatially distinct pools of TPXL-1–AurA act on kinetochore-microtubule attachments to control spindle length and stabilize bioriented attachments, thereby ensuring accurate chromosome segregation.

## RESULTS

### TPXL-1 directs AurA localization to mitotic chromatin

**Fig. 1.**
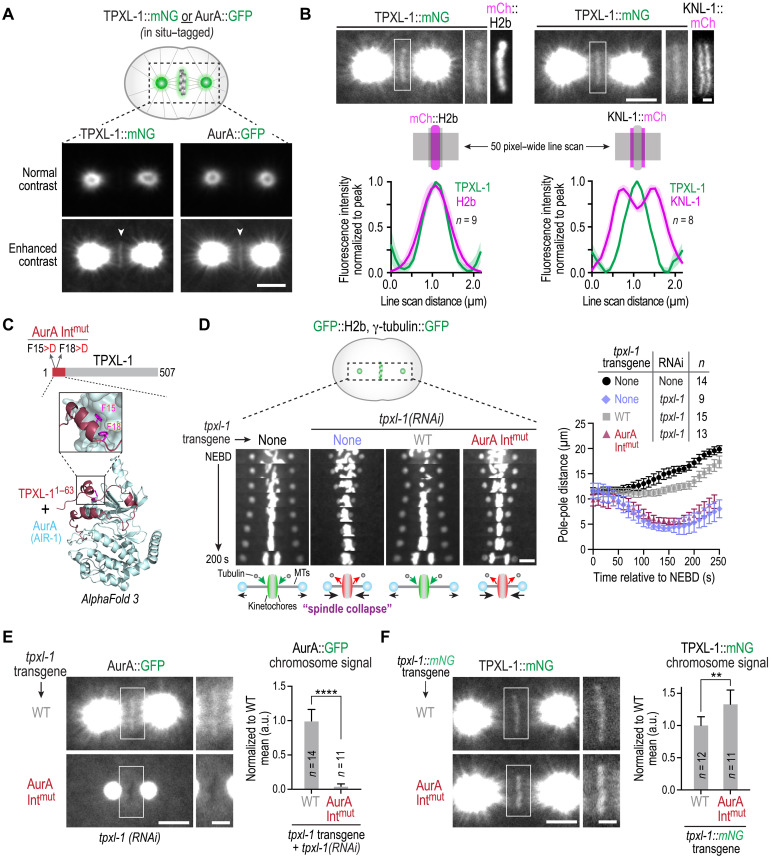
A pool of AurA is localized to chromatin via its activator TPXL-1 in one-cell *C. elegans* embryos. (**A**) Normal and enhanced contrast images of metaphase embryos. Scale bar, 5 μm. See also fig. S1A. (**B**) Top: Example images of in situ mNG-tagged TPXL-1 localization relative to mitotic chromatin (mCh::H2b) or kinetochores (KNL-1::mCh). Scale bars, 5 μm (whole spindle) and 1 μm (magnified region). Bottom: Fifty pixel–wide line scan–based measurement of fluorescence intensities. Graphs plot normalized mean signal, averaged across the indicated number of embryos (*n*). Shaded region shows the 95% confidence interval (CI). (**C**) AlphaFold 3 model of the N terminus of TPXL-1 and *C. elegans* AurA (AIR-1). Phe15 and Phe18 of TPXL-1 were mutated to disrupt the interaction. See also fig. S1, B and C. (**D**) Left: Kymographs of the spindle region in strains expressing GFP fusions that mark chromosomes (GFP::H2b) and spindle poles (γ-tubulin::GFP); the leftmost column shows a control embryo, while the other columns show TPXL-1 depleted embryos expressing indicated transgenes. Schematics below represent kinetochore state (green, polymerization-coupled; red, depolymerization-coupled) and spindle length after NEBD (MTs, microtubules). Scale bar, 5 μm. See also movie S1. Right: Graph of spindle pole separation over time for the indicated conditions. *n* is number of embryos analyzed. Error bars are SD. (**E** and **F**) Localization of AurA (E) and TPXL-1 (F) for the indicated conditions. In both (E) and (F): Left: Example images of one-cell embryos with boxed regions magnified. Scale bars, 5 μm (whole spindle) and 2 μm (magnified boxed region). Right: Graphs of chromosomal signal of AurA (E) or TPXL-1 (F) for the indicated conditions; measured values were normalized relative to the mean value of the WT condition. *n* is number of embryos analyzed. ****P < 0.0001; **P < 0.01, Mann-Whitney test. Error bars represent 95% CI. a.u., arbitrary unit.

In human cells, which have monocentric chromosomes, AurA localizes not only to spindle poles but also to the inner centromere region between sister kinetochores (*7*, *10*, *11*). In *C. elegans* embryos, which have holocentric chromosomes, AurA (AIR-1) localizes prominently to spindle poles and has also been reported to localize to chromosomes (*16*). To assess whether the mitotic AurA activator TPXL-1 exhibited a similar localization pattern as AurA, we imaged TPXL-1 and AurA fluorescently tagged at their endogenous loci. Both proteins localized robustly to spindle poles (Fig. 1A). When the image contrast was adjusted to oversaturate the pole signal, a second pool of TPXL-1 and AurA was observed on mitotic chromosomes (Fig. 1A and fig. S1A). In the *C. elegans* one-cell embryo, the 12 holocentric chromosomes each have two line-shaped kinetochores that extend along the length of the sister chromatids, forming end-coupled interfaces with ~20 microtubules (*18*). The chromatin-localized TPXL-1 signal overlapped with fluorescent histone H2b and was positioned between sister kinetochores visualized by fluorescently tagging KNL-1, a component of the outer kinetochore scaffold (Fig. 1B) (*19*). These observations identify a TPXL-1–AurA pool on the chromatin between sister kinetochores, analogous to the centromere-associated population of AurA described in human cells (*7*, *10*, *11*). Thus, the presence of both spindle pole– and chromatin-associated AurA pools appears to be a conserved feature.

To determine whether TPXL-1 or AurA mediates localization of the complex to chromosomes, we used a TPXL-1 mutant in which two phenylalanines within its N-terminal AurA-binding domain were mutated to aspartic acid (F15D;F18D). In agreement with prior work showing that these residues are required for AurA binding (*12*), AlphaFold 3 modeling predicted an interface in which the two phenylalanine residues make critical contacts with AurA (Fig. 1C and fig. S1, B and C) (*20*); we thus refer to the AurA interface-disrupting F15D;F18D mutant as AurAInt^mut^. Using a single-copy transgene insertion system that allows replacement of endogenous TPXL-1 with transgene-encoded variants (*17*), we confirmed that depleting endogenous TPXL-1 results in a characteristic spindle-collapse phenotype, which likely arises from kinetochore-microtubule attachments persisting in a depolymerization-coupled state after NEBD (Fig. 1D and movie S1). Spindle collapse was rescued by transgenic wild-type (WT) TPXL-1, but not by AurAInt^mut^ TPXL-1, which cannot bind AurA (*12*, *17*). In embryos expressing AurAInt^mut^ TPXL-1, AurA no longer localized to chromosomes (Fig. 1E), indicating that TPXL-1 recruits AurA to chromosomes. In contrast, AurAInt^mut^ TPXL-1 itself localized to chromosomes (Fig. 1F), demonstrating that TPXL-1 binds chromosomes independently of AurA. In the presence of AurAInt^mut^ TPXL-1, AurA was concentrated on the pericentriolar material matrix of the centrosome but failed to extend out from the centrosomes along spindle and astral microtubules (Fig. 1E) (*12*).

Together, these findings define a chromatin-associated pool of TPXL-1–AurA. TPXL-1 associates with chromosomes and recruits AurA to this location, where it would be well positioned to regulate kinetochore-microtubule attachments during mitosis.

### A short internal region of TPXL-1 is necessary and sufficient for chromatin localization

**Fig. 2.**
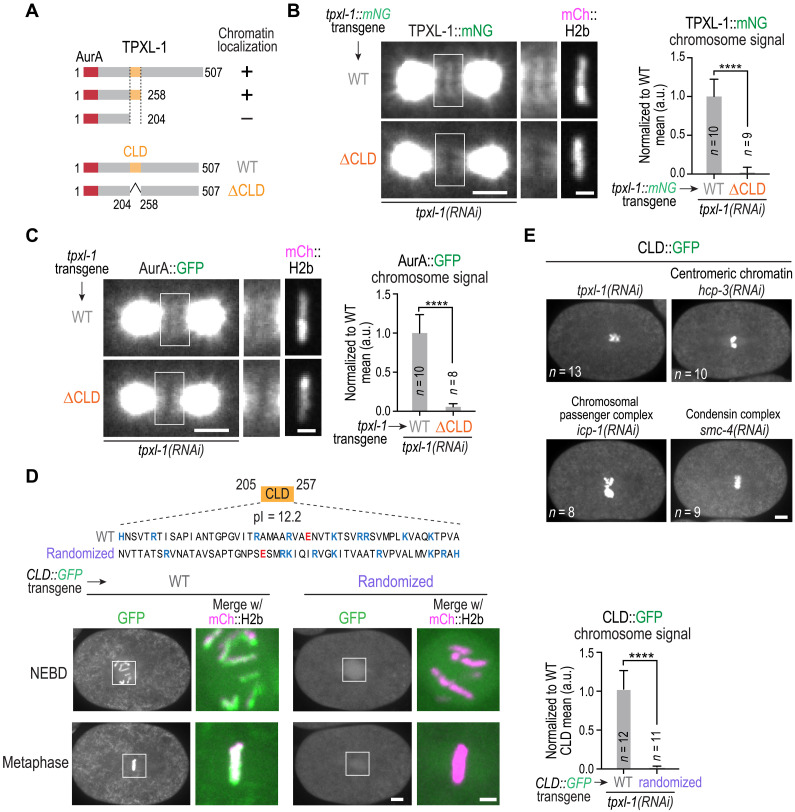
A short internal region of TPXL-1 is necessary and sufficient for chromatin localization. (**A**) Top: Summary of TPXL-1 truncation analysis identifying a CLD (amino acids 205 to 257). Bottom: TPXL-1 transgene-encoded variants used to analyze the CLD. (**B** and **C**) Localization analysis of transgene-encoded, mNG-tagged WT and ∆CLD TPXL-1 (B) or of AurA (in situ GFP-tagged) in the presence of WT or ∆CLD TPXL-1. Left: Example images of one-cell embryos for the indicated conditions, with boxed regions magnified on the right. Scale bars, 5 μm (whole spindle) and 2 μm (magnified boxed region). Right: Graphs of chromosomal signal of TPXL-1 (B) or AurA (C) for the indicated conditions. Error bars are 95% CI. *n* is number of embryos analyzed. ****P < 0.0001, Mann-Whitney test. (**D**) Top: Sequence of the 53–amino acid CLD highlighting basic (blue) and acidic (red) residues and a randomized sequence with an identical composition. Bottom: Images of embryos with transgene insertions expressing WT or randomized CLD::GFP fusions; both NEBD and metaphase stages are shown. The boxed regions are magnified on the right and shown as a merge with mCh::H2b (magenta). Scale bars, 5 μm (whole embryo) and 2 μm (magnified boxed region). See also fig. S1D. Right: Graph of chromosomal signal of WT or randomized CLD; measured values were normalized to the mean value of WT CLD. Error bars are 95% CI. *n* is the number of embryos analyzed. ****P < 0.0001, Mann-Whitney test. See also fig. S1D. (**E**) Images of one-cell embryos expressing the CLD::GFP fusion for the indicated RNAi conditions. HCP-3 is the *C. elegans* ortholog of the centromeric histone variant CENP-A; ICP-1 is the *C. elegans* ortholog of the chromosomal passenger complex subunit INCENP. *n* is the number of embryos imaged per condition. Scale bar, 5 μm.

To explore how TPXL-1–AurA associates with chromatin, we first mapped the region within TPXL-1 required for its chromatin recruitment. Since no recognizable motifs were present following the AurA-binding region at the N terminus, we used the transgene-based TPXL-1 replacement system to examine the chromatin localization of truncated variants. This analysis identified an ~50–amino acid internal region essential for chromatin localization (Fig. 2A), which we termed the chromatin localization domain (CLD). Deletion of the CLD from full-length TPXL-1 eliminated chromatin localization but did not affect spindle pole targeting (Fig. 2B). TPXL-1 lacking the CLD also failed to recruit AurA to chromatin (Fig. 2C), establishing the CLD as being required for the chromatin localization of TPXL-1 and associated AurA.

To test sufficiency, we expressed the CLD of TPXL-1 as a transgene-encoded fluorescent fusion. The isolated CLD localized robustly and specifically to chromatin, both in the presence and absence of endogenous TPXL-1, confirming that it is sufficient for chromatin association (Fig. 2, D and E, and fig. S1D). We next investigated candidate mechanisms for how TPXL-1’s CLD directs chromatin localization. Because the CLD is highly basic (isoelectric point = 12.2), we first tested whether its specific sequence or basic character was important. For this purpose, we compared the localization of the WT CLD to a sequence-randomized CLD mutant (Fig. 2D). While the randomized CLD localized diffusely in the nuclear region, no chromatin localization was observed (Fig. 2D and fig. S1D), highlighting that specific sequence motifs in the TPXL-1 CLD, rather than its overall basic character, are important for chromatin localization.

As TPXL-1 and AurA chromatin signals do not overlap with kinetochores, we did not expect CLD chromatin localization to be affected by perturbing the centromeric chromatin foundation on which kinetochores assemble. Consistent with this, the TPXL-1 CLD localized robustly to chromatin following depletion of the centromeric histone variant CENP-A (HCP-3; Fig. 2E). CLD localization was also unaffected by depletion of the chromosomal passenger complex scaffolding subunit INCENP (ICP-1) or by removal of condensin, via depletion of SMC-4. As cohesin is largely removed from mitotic chromosomes by the prophase pathway in the *C. elegans* embryo (*21*, *22*), localization of the TPXL-1 CLD to mitotic chromosomes is also unlikely to depend on cohesin. Thus, TPXL-1’s CLD binds chromatin independently of centromeric chromatin, the chromosomal passenger complex, condensin, and, likely, cohesin. These findings suggest that the CLD could directly engage chromatin itself to position a pool of TPXL-1–AurA on chromosomes to regulate kinetochore-microtubule attachments.

### The TPXL-1 CLD directly recognizes nucleosomes

**Fig. 3.**
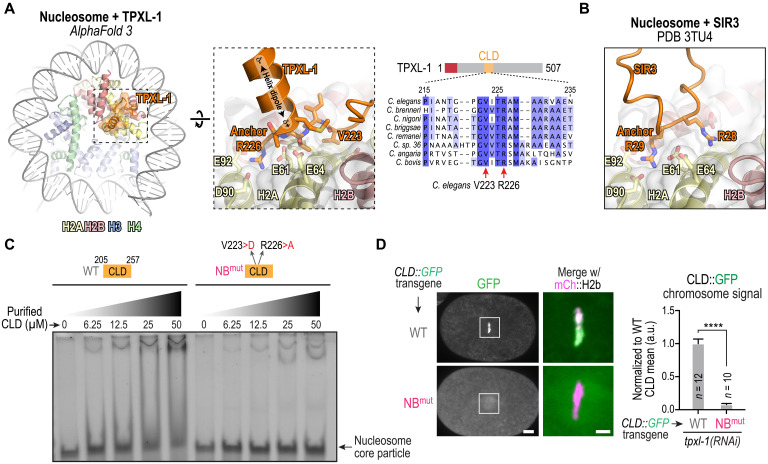
The CLD of TPXL-1 directly recognizes nucleosomes. (**A**) Left: AlphaFold 3 model of the central region of the TPXL-1 CLD (amino acids 217 to 239) and an NCP. *C. elegans* histone sequences and the Widom 601 sequence were used for the nucleosome model. The model predicts an arginine anchor mechanism for recognition of the nucleosomal acidic patch by the TPXL-1 CLD. The histone residue numbering is based on human histones. See also fig. S2. Right: Sequence alignments across related nematode species highlighting conservation of the predicted nucleosome-binding region of the TPXL-1 CLD. The two residues whose mutations are likely to disrupt the predicted interface (V223 and R226) are highlighted. (**B**) A view from the crystal structure [Protein Data Bank (PDB) 3TU4] of the interface between the bromo-associated homology domain of SIR3 and the nucleosomal acidic patch, which uses an arginine (R29) anchor. (**C**) NCP binding assays with recombinant TPXL-1 CLDs. Both WT and the predicted NB^mut^ (V223D;R226A) were expressed and purified from bacteria. NCPs were assembled using *Xenopus* histones and the Widom 601 sequence. Binding was analyzed by EMSA, using an increasing concentration gradient of WT or NB^mut^ TPXL-1 CLD. The results shown are representative of two independent experiments. (**D**) Images of embryos with transgene insertions expressing WT or NB^mut^ CLD::GFP. The boxed regions are magnified on the right and shown as a merge with mCh::H2b (magenta). Scale bars, 5 μm (whole embryo) and 2 μm (magnified boxed region). Right: Graph of chromosomal signal of WT or NB^mut^ CLD; measured values were normalized relative to the mean value of the WT CLD. Error bars are 95% CI. *n* is the number of embryos analyzed. ****P < 0.0001, Mann-Whitney test.

The prominent chromatin localization of the TPXL-1 CLD that depends on its specific sequence rather than overall basic character, along with lack of involvement of candidate localization mediators, suggested that the TPXL-1 CLD might directly recognize nucleosomal chromatin. To explore this possibility, we used AlphaFold 3 to model TPXL-1 with an octameric nucleosome core particle (NCP) (Fig. 3A and fig. S2) (*20*). A predicted structure of full-length TPXL-1 with an octameric nucleosome (fig. S2, A to C) revealed an interaction between the TPXL-1 CLD and the nucleosome acidic patch, a highly conserved surface on histones H2A and H2B that is a binding site for a range of chromatin-associated factors (*23*). Independent AlphaFold 3 predictions using the isolated TPXL-1 CLD and either an octameric nucleosome (Fig. 3A and fig. S2, D to G) or an H2A-H2B heterodimer identified the same interface with increased confidence (fig. S2, H to K). The predicted structure showed TPXL-1 residue R226 docking against several negatively charged residues on H2A (E61, E64, D90, and E92). This interaction resembles other acidic-patch binders including SIR3 (Fig. 3B) and is referred to as the “arginine anchor” (*23*). In addition to the arginine anchor, TPXL-1 residues V223 and M228 are predicted to form hydrophobic interactions with the nucleosome surface. Sequence alignments revealed that all three predicted nucleosome-binding residues (V223, R226, and M228) are conserved in *Caenorhabditis* TPXL-1 homologs (Fig. 3A).

To test whether the TPXL-1 CLD binds nucleosomes, we performed electrophoretic mobility shift assays (EMSAs) using reconstituted *Xenopus* NCPs and purified recombinant TPXL-1 CLD. Increasing concentrations of WT TPXL-1 CLD caused a concentration-dependent mobility shift of NCPs (Fig. 3C). This shift was abolished by a double mutation of the predicted nucleosome-binding residues V223D and R226A (designated as NB^mut^ for nucleosome-binding mutant). To test the functional relevance of this interaction in vivo, we generated a strain with an integrated transgene expressing a fluorescent NB^mut^ CLD fusion and analyzed localization. Unlike WT CLD, NB^mut^ CLD did not localize to chromatin (Fig. 3D). Together, these results are consistent with the TPXL-1 CLD directly binding to nucleosomes through a conserved arginine-anchor interface, and this interaction is essential for the TPXL-1 CLD to localize to chromatin in vivo.

### The chromatin-associated pool of TPXL-1–AurA is required for accurate chromosome segregation

**Fig. 4.**
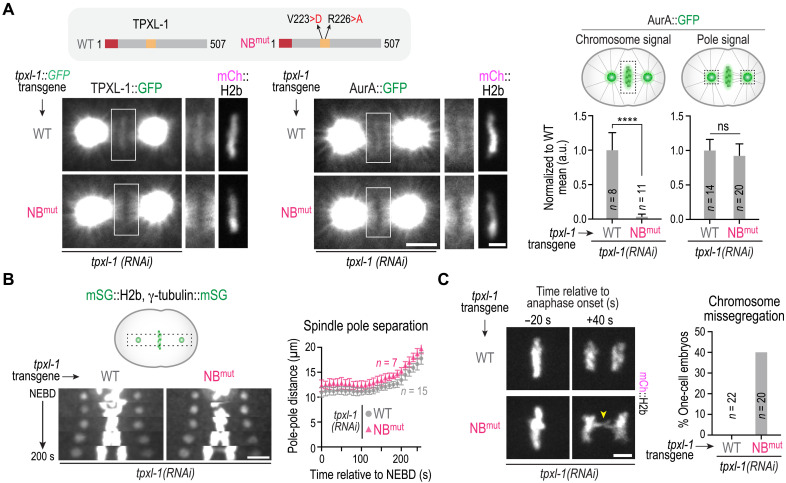
Functional consequences of selectively removing the chromatin pool of TPXL-1–AurA. (**A**) Top gray box: Schematics of WT and NB^mut^ TPXL-1 expressed from transgene insertions, either with or without a GFP tag. Bottom left: Localization of transgene-encoded TPXL-1::GFP, either WT or NB^mut^, following endogenous TPXL-1 depletion. Bottom right: Localization of AurA::GFP in the presence of untagged, transgene-encoded WT or NB^mut^ TPXL-1, following endogenous TPXL-1 depletion. The boxed regions are magnified on the right; the mCh::H2b region is only shown for the magnified panel. Scale bars, 5 μm (whole spindle) and 2 μm (magnified boxed region). Right: Graphs plotting AurA::GFP signal on chromosomes (left graph) or on spindle poles (right graph) for the indicated conditions. Measured values were normalized relative to the WT mean value. Error bars are 95% CI. *n* is the number of embryos analyzed per condition. ****P < 0.0001; not significant (ns), Mann-Whitney test. See also fig. S3A. (**B**) Left: Kymograph of spindle region of embryos expressing monomeric StayGold (mSG) fusions to label chromosomes (H2b) and spindle poles (γ-tubulin). The indicated *tpxl-1* transgene insertions were present, and endogenous TPXL-1 was depleted. Scale bar, 5 μm. Right: Graph of spindle pole separation over time for the indicated conditions. *n* is the number of one-cell embryos analyzed. Error bars are SD. See also fig. S3B. (**C**) Left: Images from time lapse movies of chromosomes in metaphase and 40 s after anaphase onset, for the indicated conditions. Scale bar, 2.5 μm. Right: Graph plotting the percentage of one-cell embryos with chromatin bridges in anaphase for the indicated conditions. See also fig. S3, C and D, and movie S2. Data shown are pooled analysis of WT and NB^mut^ TPXL-1 in mCh::H2b and mSG::H2b expressing embryos.

Having established that the TPXL-1 CLD directly recognizes nucleosomes to recruit TPXL-1–AurA to chromatin, we next asked whether disrupting the nucleosome-binding interface selectively removes TPXL-1–AurA from chromatin in vivo and whether this affects spindle assembly and chromosome segregation. Using the TPXL-1 replacement system, we expressed green fluorescent protein (GFP)–tagged and untagged versions of WT and NB^mut^ TPXL-1. NB^mut^ TPXL-1 failed to localize to chromatin but retained robust spindle pole localization (Fig. 4A and fig. S3A). Correspondingly, AurA was selectively lost from chromatin in NB^mut^ TPXL-1 embryos, while its spindle pole localization appeared unchanged (Fig. 4A). Quantitative analysis confirmed this selective depletion (Fig. 4A), indicating that the two-residue mutation that disrupts nucleosome binding by the TPXL-1 CLD removes the chromatin-associated pool of TPXL-1–AurA without affecting its spindle pole localization in vivo.

To assess the function of the chromatin pool of TPXL-1–AurA, we introduced fluorescent markers to visualize chromosomes and spindle poles and analyzed phenotypes following endogenous TPXL-1 depletion in embryos expressing transgenic WT or NB^mut^ TPXL-1. In contrast to embryos depleted of TPXL-1 with no replacement or expressing the AurA-binding mutant, which exhibited spindle collapse (Fig. 1D), transgenic NB^mut^ and ∆CLD TPXL-1 supported spindle elongation equivalently to WT TPXL-1 (Fig. 4B and fig. S3B). Thus, the chromatin-associated TPXL-1–AurA pool is not required for the transition of kinetochores out of the depolymerization-coupled state, suggesting that the spindle pole–localized population is sufficient for this function. Notably, however, elevated rates of chromosome missegregation, as measured by the frequency of anaphase chromatin bridges, were observed in both NB^mut^ and ∆CLD TPXL-1 embryos (Fig. 4C, fig. S3C, and movie S2). These bridges are characteristic of defective biorientation of the holocentric *C. elegans* chromosomes, suggesting that the chromatin pool of TPXL-1–AurA ensures chromosome biorientation on the spindle, potentially by regulating kinetochore-microtubule attachments. Together, these results suggest that distinct populations of TPXL-1–AurA carry out separable functions—the population concentrated at and emanating out from spindle poles is sufficient to promote kinetochores to exit from a depolymerization-coupled state and to regulate spindle length, and the population on chromatin ensures bioriented kinetochore-microtubule attachments.

### A short C-terminal deletion of TPXL-1 elevates the chromatin-associated pool of TPXL-1–AurA

**Fig. 5.**
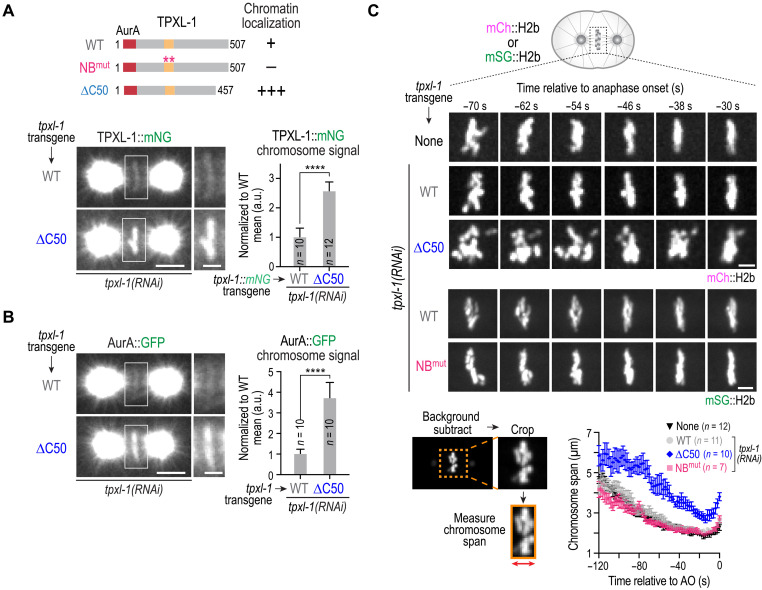
A short C-terminal deletion of TPXL-1 elevates chromatin localization and impairs chromosome alignment. (**A**) Top: Schematic summary of chromatin localization analysis of indicated TPXL-1 variants. Bottom: Example images of one-cell embryos expressing WT or ∆C50 TPXL-1::mNG following endogenous TPXL-1 depletion; the boxed regions are magnified on the right. Right: Graph of chromosomal signal of WT and ∆C50 TPXL-1; measured values were normalized relative to the mean value of the WT condition. (**B**) Analysis of AurA localization for the indicated conditions; the boxed regions are magnified on the right. Right: Graph of chromosomal AurA signal for the indicated conditions; measured values were normalized relative to the mean value of the WT condition. Scale bars in (A) and (B), 5 μm (whole spindle) and 2 μm (magnified boxed region). Error bars in graphs in (A) and (B) are 95% CI. *n* is number of embryos analyzed. ****P < 0.0001, Mann-Whitney test. (**C**) Top: Time lapse imaging of chromosome dynamics for the indicated conditions in embryos expressing either mCh::H2b (top three conditions) or mSG::H2b (bottom two conditions). Image panels are from movies that were time-aligned relative to anaphase onset (AO). Scale bars, 2.5 μm. Bottom left: Schematic of method used to measure chromosome span on the spindle over time. Bottom right: Graph plotting chromosome span over time for the indicated conditions. Error bars are the SEM. See also fig. S3E. The WT condition pools measurements from time lapse sequences obtained from mCh::H2b and mSG::H2b expressing embryos; separated WT traces for each strain background are shown in fig. S3F.

Imaging of in situ–tagged TPXL-1 and AurA revealed that while a small fraction of TPXL-1–AurA localizes to mitotic chromatin, the majority is concentrated at the spindle poles. In contrast, the isolated TPXL-1 CLD localized robustly to mitotic chromatin, indicating that the limited chromatin pool of TPXL-1–AurA is not due to a shortage of nucleosomal binding sites. Consistent with this, expression of the TPXL-1 CLD in the presence of endogenous TPXL-1 did not cause anaphase bridges (fig. S3D). These observations suggested that additional regions of TPXL-1, or its association with other structures such as spindle poles or microtubules, may restrict CLD-mediated accumulation on chromatin. We therefore asked whether the precise amount of chromatin-bound TPXL-1–AurA is important for ensuring accurate chromosome segregation. Insight into this question came from the truncation analysis of TPXL-1, which revealed that a short (50 amino acids) C-terminal deletion of TPXL-1 (∆C50) significantly elevated (by approximately three- to fourfold) the amount of TPXL-1 (Fig. 5A) and AurA (Fig. 5B) on chromosomes. Although the mechanistic basis for this enhanced chromatin association remains to be clarified, the ∆C50 mutant provided a useful tool to assess the consequences of elevating the chromatin-associated AurA pool, complementing the nucleosome-binding mutant that removes this pool.

Embryos expressing ∆C50 TPXL-1 did not exhibit spindle collapse after NEBD or anaphase chromosome bridges (fig. S3C and fig. S3E). However, analysis of chromosome behavior revealed a distinct defect compared to NB^mut^ TPXL-1 (Fig. 5C). In embryos expressing WT or NB^mut^ TPXL-1, chromosomes coalesced onto the metaphase plate and largely ceased their motion along the spindle axis over the interval between 120 and 60 s prior to anaphase onset (Fig. 5C; −120 to −60 s), followed by a small amount of additional tightening of the plate during the interval between 60 and 20 s prior to anaphase onset (Fig. 5C; −60 to −20 s). In contrast, ∆C50 TPXL-1 embryos failed to coalesce their chromosomes onto the metaphase plate during the −120 to −60-s interval. Chromosomes instead remained highly dynamic along the spindle axis, resulting in a wider, disorganized chromosome configuration that persisted through late prometaphase and metaphase (Fig. 5C, fig. S4A, and movie S3). Quantification of chromosome span [the width of the chromosome mass along the spindle axis; (*8*)] over time underscored this effect (Fig. 5C and fig. S3F). In control, WT TPXL-1, and NB^mut^ TPXL-1 embryos, chromosome span decreased from ~4.5 to ~2.5 μm during the −120 to −60-s interval as chromosomes aligned at the metaphase plate and ceased their movements along the spindle axis (Fig. 5C). In contrast, the chromosomes in TPXL-1 ∆C50 embryos exhibited a span of 4.5 μm at the end of the −120 to −60-s interval, reflecting persistent chromosome movement and incomplete congression; although a reduction in chromosome span was observed over the interval between −60 and −20 s, the chromosomes never fully congressed to form a tight metaphase plate (Fig. 5C).

Together, these findings indicate that the level of chromatin-associated TPXL-1–AurA must be precisely balanced: A low level of chromatin-associated TPXL-1–AurA is required to facilitate biorientation, likely through control of maturation of kinetochore-microtubule attachments, while excess chromatin-associated TPXL-1–AurA interferes with attachment stabilization, preventing chromosome alignment before anaphase onset.

### Elevating TPXL-1–AurA on chromatin phenocopies loss of SKA complex function

**Fig. 6.**
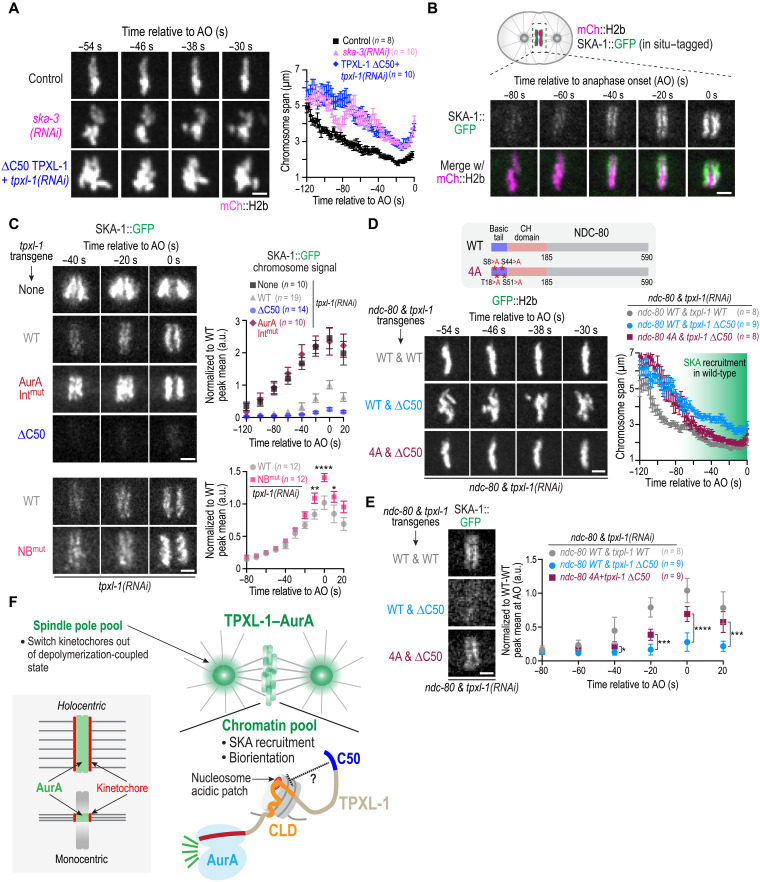
Chromatin-localized AurA regulates the Ndc80-Ska module at kinetochores. (**A**) Image sequences and quantification of chromosome span, analyzed as in Fig. 5C, for the indicated conditions. The plotted ∆C50 TPXL-1 data are the same as in Fig. 5C. The control curve is of unperturbed embryos expressing mCh::H2b. Scale bar, 2.5 μm. Error bars are the SEM. *n* is the number of embryos analyzed. See also fig. S4, A and B. (**B**) Image panels from a time lapse movie of in situ GFP-tagged SKA-1 along with mCh::H2b. Scale bar, 2.5 μm. (**C**) Left: Image panels from time lapse movies of SKA complex localization for the indicated conditions. Scale bars, 2.5 μm. Right: Quantification of chromosomal SKA-1::GFP signal over time. Error bars are 95% CI. *n* is the number of embryos analyzed. ****P < 0.0001; **P = 0.0068; *P = 0.045, Mann-Whitney test. At anaphase onset (*t* = 0), the AurAInt^mut^ and ∆C50 TPXL-1 are both significantly different from WT TPXL-1 (*P* < 0.0001). (**D**) Image panels showing chromosome behavior over time for the indicated conditions; graph on the right plots chromosome span over time, measured as in Fig. 5C. *n* is number of embryos analyzed; error bars are SEM. Scale bar, 2.5 μm. See also fig. S4C. The NDC-80 WT and 4A conditions are significantly different between −50 and −4 s (*P* values ranged from <0.0001 to <0.01 in two-tailed unpaired *t* test). (**E**) Analysis of SKA complex localization for the indicated conditions. Image panel shown is just prior to anaphase. Scale bar, 2.5 μm. Graph on the right plots chromosomal SKA-1::GFP signal over time. Error bars are 95% CI; *n* is number of embryos analyzed. *P = 0.0142 at −40 s; ***P = 0.0008 at −20 s; ****P < 0.0001 at 0 s; ***P = 0.0003 at 20 s, Mann-Whitney test comparing NDC-80 WT and 4A conditions. (**F**) Schematic summary of key findings. See text for details.

The delayed alignment and persistent chromosome motion observed in ∆C50 TPXL-1 embryos, which have elevated levels of TPXL-1–AurA on chromatin, resembled the phenotype caused by inhibiting the microtubule-binding SKA complex (*8*). In *C. elegans*, the SKA complex is recruited in an NDC-80 complex-dependent manner and reduces the dynamicity of kinetochore-microtubule attachments to align chromosomes prior to anaphase onset (*8*). To compare the ∆C50 TPXL-1 and SKA complex loss phenotypes directly, we quantified chromosome span over time using SKA-3 depletion, which causes loss of kinetochore localization of the microtubule-binding SKA-1 subunit of the *C. elegans* SKA complex (fig. S4B). Chromosomes in ∆C50 TPXL-1 and *ska-3*(*RNAi*) embryos exhibited overall similar behavior (Fig. 6A, fig. S4A, and movie S3).

Consistent with prior work (*8*), imaging of in situ–tagged SKA-1 revealed that it normally becomes prominent at kinetochores during a short window before anaphase, coinciding with dampened chromosome motion and tight metaphase plate formation (Fig. 6B). We next examined the effect of ∆C50 TPXL-1 on SKA-1 recruitment. As shown previously (*24*), TPXL-1 depletion or mutation of its AurA binding site caused hyper-recruitment of SKA to chromosomes (Fig. 6C). Expression of WT TPXL-1 restored SKA-1 recruitment to normal levels, whereas ∆C50 TPXL-1 significantly reduced SKA localization to kinetochores (Fig. 6C), which likely explains the persistent chromosome movement and incomplete alignment observed when chromosomal AurA is elevated. To complement this analysis, we also analyzed the effect of removing the chromatin-associated TPXL-1–AurA pool using NB^mut^ TPXL-1. In this case, a modest but significant increase in SKA complex recruitment was observed (Fig. 6C), suggesting that loss of the chromatin-associated TPXL-1–AurA pool enhances SKA loading, potentially leading to premature stabilization of erroneous attachments and the observed chromosome missegregation. The lower extent of SKA over-recruitment seen with the NB^mut^ TPXL-1 compared with TPXL-1 depletion or loss of TPXL-1 AurA binding (Fig. 6C) suggests that TPXL-1–AurA emanating out from the spindle poles also restrains SKA recruitment to kinetochores. Together, these findings indicate that the chromatin-associated pool provides an additional layer of SKA recruitment control that is important to biorient all chromosomes.

The observation that chromatin-associated TPXL-1–AurA negatively regulates SKA complex recruitment raised the question of which AurA target sites mediate this effect. Prior work has shown that mutating four Aurora kinase phosphorylation sites in the basic N-terminal tail of Ndc80 (Ndc80 4A mutant) increases SKA complex recruitment (*8*). To test whether phosphorylation at these sites is required for the chromatin-associated TPXL-1–AurA pool to exert its effect on chromosome dynamics, we generated strains combining the transgenes expressing WT or ∆C50 TPXL-1 with transgenes expressing WT or nonphosphorylatable 4A NDC-80 and analyzed chromosome dynamics following endogenous TPXL-1 and NDC-80 depletion. When NDC-80 4A was present, the persistent chromosome movement and incomplete congression as well as the reduced SKA complex recruitment caused by ∆C50 TPXL-1 were significantly rescued (Fig. 6, D and E, and fig. S4C). These results are consistent with chromatin-associated TPXL-1–AurA regulating SKA complex recruitment and chromosome dynamics through phosphorylation of the NDC-80 N-terminal tail.

We note that, in the −100 to −60-s interval relative to anaphase onset, SKA depletion exhibited transient but unstable chromosome alignment whereas ∆C50 TPXL-1 did not (Fig. 6A). In addition, NDC-80 4A did not suppress the wider chromosome span observed with ∆C50 TPXL-1 prior to −70 s, around the time when SKA recruitment ramps up (Fig. 6D). These observations suggest that the elevated chromatin-associated AurA in ∆C50 TPXL-1 may also perturb an SKA-independent chromosome alignment pathway in early prometaphase.

Overall, the above data indicate that the chromatin pool of TPXL-1–AurA controls SKA complex recruitment to stabilize kinetochore-microtubule attachments, with a precise level of TPXL-1–AurA activity at this location being critical to ensure biorientation of all chromosomes prior to anaphase onset.

## DISCUSSION

Mitotic kinases of the Aurora, Plk (Polo-like kinase), and Cdk (cyclin-dependent kinase) families act on hundreds of substrates in distinct cellular locations to ensure that the replicated genome is accurately distributed to daughter cells. A major challenge with investigating mitotic kinase functions is their breadth of action, which leads to complex phenotypes following kinase activity inhibition. Here, we focus on AurA, a multifunctional kinase present in different regulator-bound pools concentrated at diverse cellular locations. Our analysis focused on the AurA populations associated with TPXL-1, an ortholog of TPX2, which concentrates at spindle poles and on chromatin and is a major regulator of spindle assembly and chromosome segregation. Our findings reveal that spatially distinct pools of TPXL-1–AurA perform complementary functions in regulating kinetochore-microtubule attachments that in turn affect spindle length and chromosome biorientation. The spindle pole–associated TPXL-1–AurA population promotes the transition of kinetochores out of a depolymerization-coupled state following NEBD, enabling the proper regulation of spindle length. The chromatin-associated pool functions as a brake on SKA complex recruitment, enabling resolution of attachment errors and full biorientation (Fig. 6F). While the pool at spindle poles also restrains SKA recruitment, we suggest that the chromatin pool provides a second layer of control that is critical to ensure biorientation as chromosomes move away from the poles on their path to alignment on the metaphase plate.

Our ability to address the functions of distinct TPXL-1–AurA pools was facilitated by the elucidation of the mechanism by which it is recruited to chromatin. We found that TPXL-1 directly recognizes nucleosomes, likely using an arginine anchor mechanism to engage the nucleosome acidic patch (Fig. 6F). This mechanism of nucleosome recognition is widespread and has been characterized in many diverse factors that engage with chromatin (*23*). Engineering mutations predicted on the basis of structural modeling to disrupt nucleosome recognition by TPXL-1 abrogated nucleosome binding in vitro and chromatin localization in vivo. Removal of the chromatin pool of TPXL-1–AurA did not cause spindle collapse, indicating that the spindle pole pool is sufficient to switch kinetochores out of a depolymerization-coupled state and allow assembly of a normal length spindle. Instead, loss of the chromatin pool resulted in anaphase chromosome bridges. In the *C. elegans* embryo, which have holocentric chromosomes, anaphase bridges are characteristic of biorientation defects where the kinetochore of a single chromatid connects to microtubules from both spindle poles. Notably, the loss of the chromatin pool elevated SKA complex recruitment, suggesting that incorrect kinetochore-microtubule attachments are prematurely stabilized, leading to the anaphase defects. Conversely, analysis of a mutant that elevated the TPXL-1–AurA chromatin pool prevented SKA complex recruitment, resulting in a kinetochore-attachment stabilization defect and hyperdynamic chromosome movement on the spindle. In the *C. elegans* embryo, the cessation of chromosome movement coincident with alignment to the equator is dependent on SKA recruitment by the NDC-80 complex (*8*). In this system, there is no equivalent of the Astrin-SKAP complex that lubricates the bioriented kinetochore-microtubule interface in human cells (*25*); consequently, chromosome behavior in the *C. elegans* embryo resembles what is observed in human cells lacking Astrin-SKAP.

The effects of loss of SKA function are observed during the interval between −120 and −60 s prior to anaphase onset, when levels of SKA on chromosomes are relatively low, suggesting that biorientation and alignment are mediated by low-level SKA recruitment. A major wave of SKA recruitment occurs after chromosome alignment during the 60-s interval immediately prior to anaphase onset; this likely represents a second step in attachment stabilization that prepares kinetochores to withstand pulling forces after cohesion is lost in anaphase. A question raised by these observations is how a small, constant level of TPXL-1–AurA associated with chromatin precisely regulates SKA recruitment so that stabilization is coupled to the success of chromosomes in achieving a bioriented state. As SKA recruitment is regulated by NDC-80 phosphorylation, we speculate that the dynamic recruitment or regulation of phosphatase activities may play a key role in sensing biorientation. One possibility is that phosphorylation of targets like the N-terminal tail of NDC-80 by chromatin-bound TPXL-1–AurA holds kinetochore-microtubule attachments in a dynamic state until the threshold set by this phosphorylation is overcome by phosphatases recruited in response to biorientation and alignment. One of the major phosphatases at kinetochores is Protein Phosphatase 1 (PP1), which is recruited to its docking site on the KNL-1 kinetochore scaffold during this interval (*26*). However, loss of KNL-1 docked PP1 does not cause the persistent hyperdynamic chromosome movement phenotype (*19*), suggesting that regulation of TPXL-1–AurA activity or of other phosphatases may also contribute to coupling kinetochore-microtubule attachment stabilization to biorientation.

TPXL-1 is divergent from TPX2, its vertebrate ortholog, and chromatin association of TPX2 has, to our knowledge, not been reported. AlphaFold 3 modeling also does not predict interaction of human TPX2 with nucleosomes. However, multiple studies in human cultured cells have reported AurA targeting to the inner centromere, the chromatin region between the sister kinetochores on monocentric chromosomes, and association of AurA with chromosomal passenger complex subunits has been suggested to mediate this localization (*7*, *10*, *11*). In addition, functional studies have implicated AurA in spindle length control and kinetochore-microtubule attachment regulation (*9*–*11*, *13*, *14*, *27*). Thus, while the precise mechanism of chromatin targeting may differ, in both holocentric *C. elegans* and monocentric human cells, AurA activity is concentrated on the chromatin adjacent to kinetochore-microtubule attachments (Fig. 6F). We speculate based on our results that, across this large evolutionary distance, AurA is present at this site because the balance between local AurA and opposing phosphatase activities controls the maturation of kinetochore-microtubule attachments via phosphoregulated SKA complex recruitment, which in turn stabilizes the bioriented configuration prior to anaphase onset.

## MATERIALS AND METHODS

### *C. elegans* strains

*C. elegans* strains used in the study are listed in table S1. All strains were maintained on Nematode Growth Media (NGM) plates seeded with OP50 *Escherichia coli* to produce a feeding lawn. Hermaphrodites were periodically passed to fresh plates to prevent starvation and stored at 20°C.

All RNA interference (RNAi)–resistant transgenes were generated by single-copy insertions at specific chromosomal loci using the MosSCI method (*28*). Transgene insertions expressing TPXL-1 WT and AurA binding mutants, tagged with monomeric NeonGreen (mNG) or untagged, were previously described (*17*). All other TPXL-1 mutants used in this study were generated by injecting both gonad arms of EG6429 young adults with pCFJ350 integration plasmids containing the TPXL-1 mutations indicated in each figure. Gene blocks containing the nucleosome binding mutants (V233D;R226A) were purchased from IDT and Gibson cloned into pCFJ350. TPXL-1::GFP fusions contain GFP with introns enriched for periodic An/Tn clusters to prevent transgene silencing [referred to as GFP(PATC-enriched)] (*29*). NDC-80 RNAi-resistant strains were previously described (*8*).

Integration plasmids (pCFJ350) were mixed with coinjection markers pCFJ190, pCFJ104, pGH8, and pMA122 to screen against extrachromosomal arrays, along with a transposase (pCFJ601). Injected worms were singled onto NGM plates and grown for 7 to 10 days before heat shock was performed for 3 hours at 34°C. Surviving progeny were screened for the absence of array markers (mCherry) and singled onto fresh plates. Successful integration was assessed by polymerase chain reaction (PCR), and integrated cassettes were sequence-validated using Oxford Nanopore longread sequencing.

Spindle poles and chromosomes were tagged using monomeric StayGold (mSG) (*30*, *31*) fused to the C terminus of *tbg-1* and to the N terminus of *his-11* in the same operon, separated by an operon linker. The operon cassette was then integrated on chromosome I using the MosSCI method.

In situ GFP-tagged *ska-1* was previously described (*8*). Endogenous *tpxl-1* was tagged at the C terminus using CRISPR-Cas9 (*32*). A Cas9-ribonucleoprotein mix, containing the guide sequence (5′-CCCGGGCACTGCTTCGA-3′), repair template, Cas9 (purchased from Berkley MacroLab), and selection markers, was injected into young N2 adult hermaphrodites. After 3 to 5 days, successfully edited strains were validated using genotyping PCR and Sanger sequencing of the edited genomic region.

### RNA interference

Double-stranded RNA (dsRNA) was produced by amplifying DNA templates from *C. elegans* cDNA or genomic DNA using primers containing the T7 and T3 promoter sequences listed in table S2 for PCR. Templates were purified using a QIAquick PCR Purification Kit (QIAGEN). Single-stranded RNA was generated from DNA templates using MEGAscript T3 and T7 Transcription Kits (Invitrogen) and purified using a MEGAclear Transcription Clean-Up Kit (Invitrogen). Single-stranded RNA was subsequently annealed at 68°C for 10 min followed by 37°C for 30 min to produce final dsRNA products. L4 hermaphrodites were injected with final dsRNA products. For tpxl-1(*RNAi*), injected animals were incubated at 16°C for 24 hours, followed by a shift to 20°C for 24 hours before imaging. L4 hermaphrodites injected with *hcp-3*, *icp-1*, and *smc-4*(*RNAi*) (Fig. 2E) and *ska-3*(*RNAi*) (Fig. 6A) were incubated at 20°C for 38 to 46 hours before dissection and imaging. Co-depletion of endogenous *tpxl-1* and *ndc-80* (Figs. 6D and 5E) was performed by mixing each dsRNA at 1:1 ratios and incubating L4 hermaphrodites at 20°C for 48 hours before imaging.

### Fluorescence imaging of *C. elegans* embryos

Embryos were dissected from adult hermaphrodites into M9 buffer and mounted on a 2% agarose pad using a mouth pipette. Embryos were then covered with a 22 mm–by–22 mm coverslip (no. 1.5), and all imaging was performed in a room cooled to 20°C. Spindle pole separation, chromatin localization, and kinetochore recruitment assays were imaged on a Nikon Ti2 confocal system with a Yokogawa CSU-X1 confocal scanner and an Andor iXon electron multiplication back-thinned charge-coupled device (EMCCD) camera (Fig. 1D and fig. S3, B and D) or a Kinetix 22 active-pixel sensor [complementary metal-oxide semiconductor (CMOS)] (Fig. 4B) using a 60× 1.42 NA (numerical aperture) Plan Apochromat Lens (Nikon). A 7 × 2 μm *z*-stack was collected every 10 s for spindle pole separation, or every 20 s for fluorescence localization assays.

To assay chromosome dynamics, one-cell embryos expressing mCherry::H2b, GFP::H2b, or mSG::H2b were imaged on either the Nikon Ti2 (GFP::H2b and mSG::H2b), or with a Zeiss Axio Observer Z1 microscope equipped with a Yokogoawa CSU-X1 spinning disk and a QuantEM:512SC (Photometrics) EMCCD with a 60× 1.4 NA Plan Aprochromat Lens (Zeiss) using ZEN Blue microscopy software (Zeiss). High-speed chromosome dynamics (chromosome span) were imaged with 6 × 2 μm *z*-stacks every 2 s.

Embryos (Figs. 1B and 6C) were also imaged on an Eclipse Ti2-E equipped with a CSU-W1 SoRA (Yokogawa) spinning disk head, a 60× 1.49 NA Plan Aprochromat Lens (Nikon), and dual Prime 95B CMOS cameras (Kinetix) at 12-bit settings using NIS-Elements software (Nikon). Images were acquired every 20 s using 7 × 2 μm *z*-stacks.

### Image analysis

ImageJ (Fiji) was used to process all microscope images. For spindle pole separation analysis, maximum intensity projections of GFP- or mSG-tagged *tbg-1* and *his-11* were time-aligned to NEBD, which was defined as the frame when the diffuse nuclear histone signal equalized with the cytoplasm. Spindle length was then measured as the distance between the two centrosomes over time using ImageJ.

Line scans of mNG-tagged *tpxl-1* in combination with mCherry-tagged *his-11* or *knl-1* were performed on maximum intensity projections of the four *z*-slices centered through the metaphase plate at −20 s relative to anaphase onset. Anaphase onset was defined as the first time point in which chromosomes could be distinguished as two separate masses based on HIS-11 and TPXL-1 signals. A 50 pixel–wide line scan was drawn perpendicular to the long axis of the metaphase plate and centered on the chromosome midline. The fluorescence of the line was then measured by plotting the profile in ImageJ. Local background was then measured by extending the line by 2 μm and subtracted from the fluorescence intensity profile. Background-subtracted values were then normalized to the peak intensity (the highest of the two peaks in KNL-1::mCherry) and plotted as a function of distance along the line scan.

Kinetochore and chromatin recruitment assays were performed on maximum intensity projections of the four *z*-slices fully encompassing the chromosome signal. A box was traced around chromosomes and kinetochores to measure chromatin and kinetochore signal, respectively, and integrated density was recorded using Image J. Local background was calculated by extending the box by five pixels on all sides and subtracting the integrated density and area from the initial box. The integrated chromatin or kinetochore signal was then calculated by subtracting the background signal. This was repeated every 20 s through anaphase onset for endogenously labeled SKA-1 dynamics, and 20 s prior to anaphase onset for TPXL-1 and AIR-1 measurements.

Chromosome segregation analysis was performed on maximum intensity projections of mSG::H2b, mCherry::H2b, or GFP::H2b signals. Segregation errors were scored if the fluorescence histone signal was visible between separating sister chromatids for at least 20 s after anaphase onset.

Chromosome span was measured in movies that were filmed with 2-s intervals at 7 × 2 μm *z*-stacks for mSG::H2b and mCherry::H2b. Stacks were maximum-projected, background-subtracted, and converted to 8-bit in ImageJ. A minimum bounding box was then fit to the edge of the H2b fluorescence signal (the boundary where the pixel value is 0), and the width of the bounding box was measured at every 2-s interval during chromosome congression between NEBD and anaphase onset.

### Protein expression and purification

Codon-optimized gene blocks encoding the CLD (residues 205 to 257) of TPXL-1 (WT and V233D;R226A) were purchased from GenScript and cloned into the UC Berkeley MacroLab vector 2C-T (Addgene, no. 29706), which encodes an N-terminal TEV protease–cleavable His6-maltose binding protein (MBP) tag. Plasmids were transformed into Rosetta 2 (DE3) pLysS *E. coli* competent cells (EMD Millipore), and overnight cultures were used to inoculate 1-liter cultures of 2XYT media at 37°C with shaking at 180 rpm. At OD_600_ (optical density at 600 nm) of 0.5, protein expression was induced with the addition of 0.2 mM isopropyl β-d-1-thiogalactopyranoside, and the cultures were shifted to 20°C and incubated for an additional 16 hours. Cells were harvested by centrifugation, resuspended in lysis buffer (20 mM Hepes-NaOH, pH 7.5; 300 mM NaCl; 10% glycerol; 5 mM imidazole; and 5 mM β-mercaptoethanol), and lysed by sonication (Branson Sonifier), and the cell lysate was clarified by centrifugation. The supernatant was loaded onto a nickel affinity column (QIAGEN Ni-NTA Superflow) in lysis buffer, washed with 20 mM Hepes-NaOH, pH 7.5; 300 mM NaCl; 10% glycerol; 5 mM imidazole; and 5 mM β-mercaptoethanol, and eluted in 20 mM Hepes-NaOH, pH 7.5; 300 mM NaCl; 10% glycerol; 500 mM imidazole; and 5 μM betamercaptoethanol. Fractions were pooled and diluted to 100 mM NaCl using 20 mM Hepes-NaOH, pH 7.5; 10% glycerol; and 5 mM β-mercaptoethanol before loading onto an anion-exchange column (HiTrap Q HP, no. 17-1154-01, Cytiva). Bound proteins were eluted with a linear gradient from 100 mM to 1 M NaCl. Pooled fractions were then run on a size-exclusion column (Superdex 200 16/600, Cytiva), concentrated, and stored at −80°C.

### Nucleosome binding assays

NCPs were reconstituted following published protocols (*33*). Briefly, lyophilized *Xenopus laevis* histones H2A, H2B, H3, and H4 were purchased from the Histone Source at Colorado State University (https://histonesource-colostate.nbsstore.net/). Histones were unfolded with incubation and shaking for 1 hour at room temperature in unfolding buffer [6 M guanidine hydrochloride; 20 mM tris-HCl, pH 7.5; and 5 mM dithiothreitol (DTT)] and mixed in equimolar ratio for 1 mg/ml final concentration. Histones were refolded into an octamer by dialysis in refolding buffer (2 M NaCl; 10 mM tris-HCl, pH 7.5; 1 mM EDTA; and 5 mM β-mercaptoethanol). After refolding, histones were concentrated via centrifugation and loaded onto a size-exclusion column (Superdex 200) equilibrated in refolding buffer. Fractions were analyzed by SDS–polyacrylamide gel electrophoresis, and fractions containing pure histones were pooled. For nucleosome reconstitution, the Widom 601 DNA sequence was amplified by PCR, purified, and concentrated. DNA was added to the histone octamer in 1:1.2 molar ratio and dialyzed in 1.4 M KCl; 10 mM tris-HCl, pH 7.5; 0.1 mM EDTA; and 1 mM DTT for 1 hour at 4°C. Low-salt buffer (10 mM KCl; 10 mM tris-HCl, pH 7.5; 0.1 mM EDTA; and 1 mM DTT) was slowly pumped into the high-salt buffer for a couple of hours and then replaced with low-salt buffer and dialyzed overnight. Nucleosomes were concentrated with a centrifugal filter and injected onto a size-exclusion column (Superose 6) in 20 mM Hepes pH 7.5; 20 mM NaCl; 0.5 mM EDTA; and 1 mM DTT. Fractions were analyzed on a 6% acrylamide tris-borate EDTA (TBE) gel (prerun in 0.5× TBE for 150 V for 1 hour at 4°C) run for 1 hour at 150 V at 4°C. The gel was stained with SYBR Gold (S11494, Invitrogen; 1:10,000 dilution), imaged with a ChemiDoc system, and pure nucleosomes were pooled and concentrated with a centrifugal filter.

For the EMSA analysis, recombinant, purified His6-MBP-TPXL-1 CLD (WT or NB^mut^) and 25 nM reconstituted NCPs were incubated for 15 min at room temperature in binding buffer (20 mM Hepes, pH 7.5; 25 mM NaCl; 10% glycerol; and 0.5 mM β-mercaptoethanol). After adding sucrose to 5% final concentration, samples were loaded onto a 6% TBE gel, prerun at 150 V for 1 hour at 4°C, and run for 80 min at 120 V at 4°C in 0.2× TBE. The gel was then incubated in SYBR Gold for 10 min in the dark. Gel was imaged on a ChemiDoc Imaging System (12003153, ChemiDoc).

### AlphaFold 3 structure predictions

Structure predictions of TPXL-1 and the NCP or an H2A-H2B dimer was performed using AlphaFold 3 (*20*). Predicted Aligned Error (PAE) plots were generated using the PAEViewer web server (*34*). The *C. elegans* core histone sequences used in the model are HIS-3 (H2A), HIS-29 (H2B), HIS-2 (H3), and HIS-67 (H4); note that there are multiple genes encoding 100% identical core histone sequences in the *C. elegans* genome (16 genes for H2A, 15 genes for H3, and 16 genes for H4; for H2B, there are five genes encoding 100% identical sequence to HIS-3, and six genes encoding H2Bs with one additional amino acid, an alanine, in position 2). Structural analysis and depictions were carried out in PyMOL (*35*).

### Statistical analyses

Statistical analysis was performed with Prism 10 software (GraphPad). Statistical significance of fluorescence intensity averages was determined by a two-sided Mann-Whitney test. Definitions for *P* values are given in the corresponding figure legends. Error bars for pole-pole distance measurements represent standard deviations. Error bars for fluorescence intensities are 95% confidence intervals (CIs). Error bars for chromosome span analysis represent standard error of the mean. Each experiment represents (*n*) number of embryos from a minimum of five different hermaphrodites per condition.

## Supplementary Materials

### The PDF file includes:

Figs. S1 to S4 Tables S1 and S2 Legends for movies S1 to S3 References
