# Supplementary material for: A chromatin-associated pool of Aurora A controls kinetochore-microtubule attachments to ensure chromosome biorientation
Source: Sci Adv. 2026 May 6;12(19):eaed5283. doi: 10.1126/sciadv.aed5283 (PMC13148335; doi:10.1126/sciadv.aed5283)
Supplement: Supplementary file 1 — Figs. S1 to S4 Tables S1 and S2 Legends for movies S1 to S3 References [file sciadv.aed5283_sm.pdf]

Science Advances
AAAS

# Supplementary Materials for

## A chromatin-associated pool of Aurora A controls kinetochore-microtubule attachments to ensure chromosome biorientation

Johnathan L. Meaders *et al.*

Corresponding author: Johnathan L. Meaders, jlmeaders@ucsd.edu; Arshad Desai, abdesai@ucsd.edu

*Sci. Adv.* **12**, eaed5283 (2026)
DOI: 10.1126/sciadv.aed5283

**The PDF file includes:**

Figs. S1 to S4
Tables S1 and S2
Legends for movies S1 to S3
References

**Other Supplementary Material for this manuscript includes the following:**

Movies S1 to S3

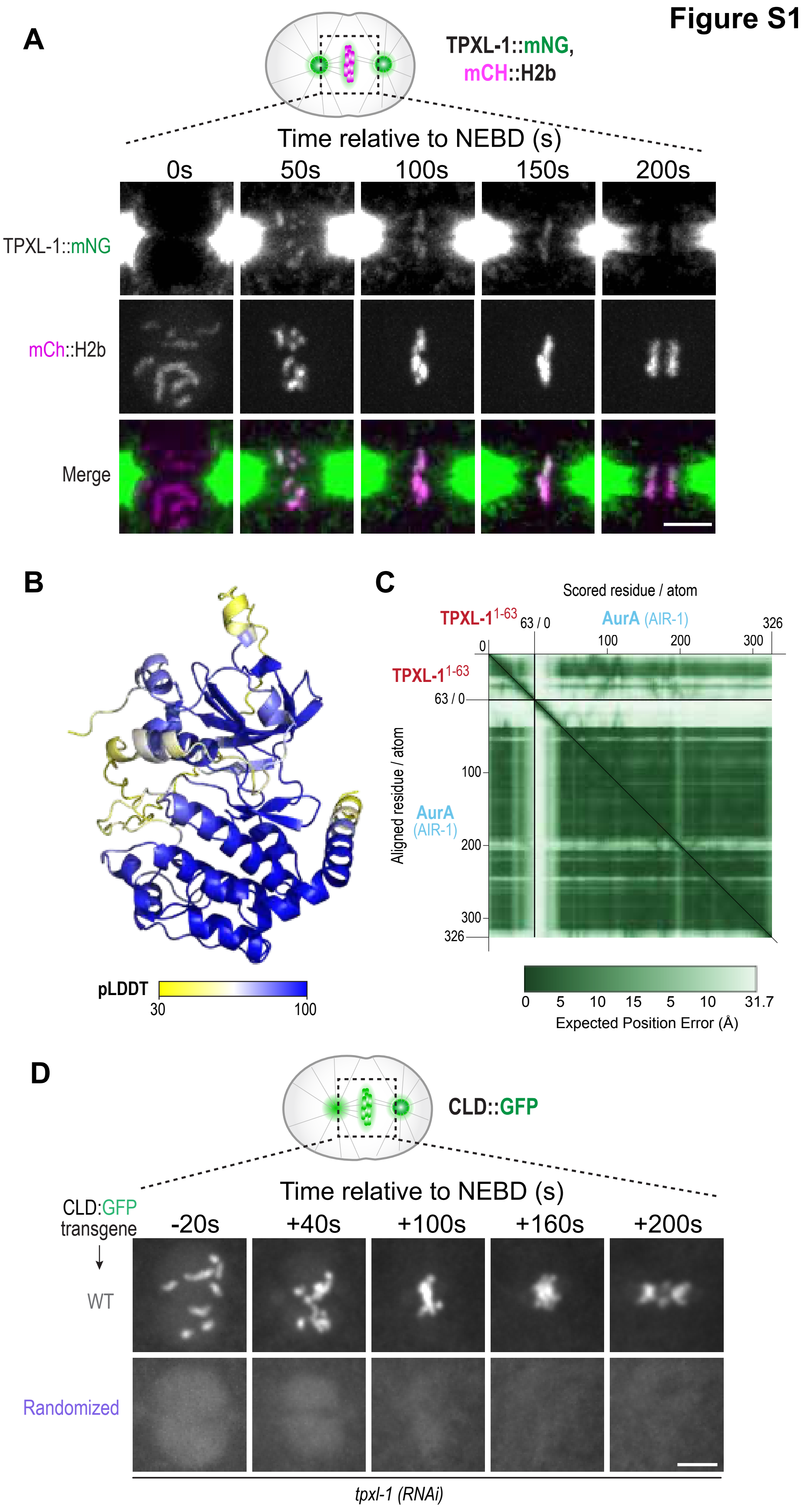
Figure S1
A
TPXL-1::mNG,
mCH::H2b
Time relative to NEBD (s)
0s
50s
100s
150s
200s
TPXL-1::mNG
mCh::H2b
Merge
B
pLDDT
30
100
C
Scored residue / atom
TPXL-1^1-63
63 / 0
AurA (AIR-1)
326
0
100
200
300
Aligned residue / atom
326
0
5
10
15
5
10
31.7
Expected Position Error (Å)
D
CLD::GFP
Time relative to NEBD (s)
CLD:GFP transgene
-20s
+40s
+100s
+160s
+200s
WT
Randomized
tpxl-1 (RNAi)

**Fig. S1. Alphafold model of TPXL-1–AurA interface and chromatin localization of TPXL-1::mNG over time. (A)** Image panels from a timelapse movie of TPXL-1::mNG in an embryo co-expressing mCh::H2b. Contrast was enhanced in the TPXL-1::mNG channel to visualize the chromatin pool. Scale bar, 5 µm. **(B)** AlphaFold3 predicted structure of TPXL-$1^{1\text{-}63}$ and AurA (AIR-1), colored by confidence (pLDDT: predicted local distance difference test). **(C)** AlphaFold3 predicted aligned error (PAE) plot for TPXL-$1^{1\text{-}63}$ and AurA (AIR-1) model, generated using the PAEViewer server. **(D)** Image panels from timelapse movies of WT and Randomized TPXL-1 CLD, following endogenous TPXL-1 depletion. Scale bar, 5 µm. Image sequences in (*A*) and (*D*) were time-aligned relative to nuclear envelope breakdown (NEBD).

# Figure S2

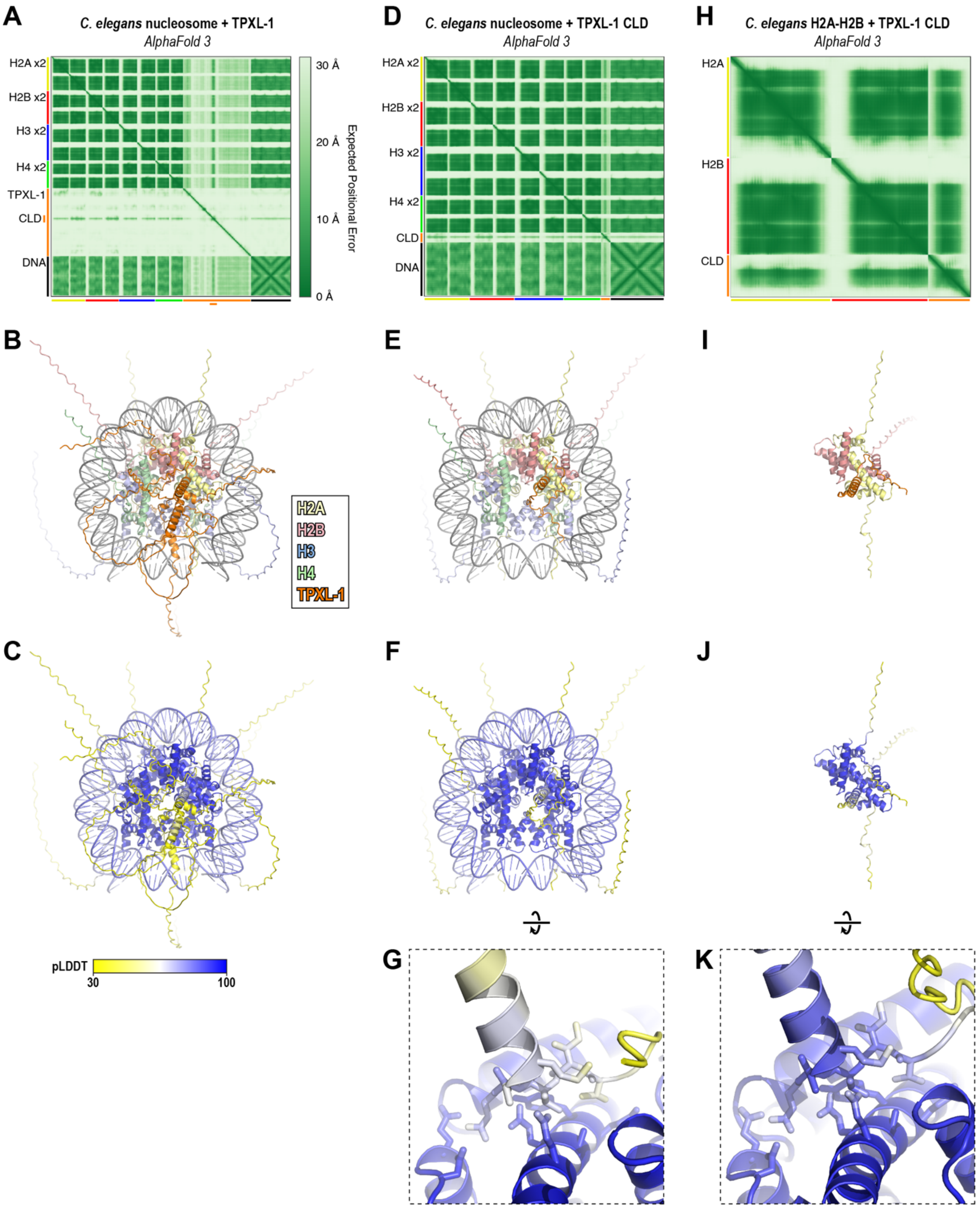
A
C. elegans nucleosome + TPXL-1
AlphaFold 3
H2A x2
H2B x2
H3 x2
H4 x2
TPXL-1
CLD
DNA
30 Å
20 Å
10 Å
0 Å
Expected Positional Error
D
C. elegans nucleosome + TPXL-1 CLD
AlphaFold 3
H2A x2
H2B x2
H3 x2
H4 x2
CLD
DNA
H
C. elegans H2A-H2B + TPXL-1 CLD
AlphaFold 3
H2A
H2B
CLD
B
E
I
H2A
H2B
H3
H4
TPXL-1
C
F
J
pLDDT
30
100
G
K

**Fig. S2. Alphafold3 models of TPXL-1 and the TPXL-1 CLD with the nucleosome core particle or an H2A-H2B dimer. (A)** AlphaFold 3 predicted aligned error (PAE) plot for a prediction of a *C. elegans* nucleosome octamer (two copies each of histones H2A, H2B , H3, and H4) with full-length TPXL-1 and a double-stranded DNA (Widom 601 sequence). The *C. elegans* core histone sequences employed in the model are: H2A (HIS-3), H2B (HIS-29), H3 (HIS-2), H4 (HIS-67); note that there are multiple genes encoding 100% identical core histone sequences in the *C. elegans* genome. **(B)** AlphaFold 3 predicted structure of a *C. elegans* nucleosome octamer with full-length TPXL-1 and Widom 601 DNA. Histones are colored yellow (H2A), pink (H2B), blue (H3), and green (H4), and TPXL-1 is colored orange. **(C)** AlphaFold 3 predicted structure of a *C. elegans* nucleosome octamer with full-length TPXL-1 and Widom 601 DNA, colored by confidence (pLDDT: predicted local distance difference test). **(D)** AlphaFold 3 predicted aligned error (PAE) plot for a prediction of a *C. elegans* nucleosome octamer with the TPXL-1 CLD (aa 217-239) and a double-stranded DNA (Widom 601 sequence). **(E)** AlphaFold 3 predicted structure of a *C. elegans* nucleosome octamer with the TPXL-1 CLD and Widom 601 DNA, colored as in panel (*B*). **(F)** AlphaFold 3 predicted structure of a *C. elegans* nucleosome octamer with the TPXL-1 CLD and Widom 601 DNA, colored by confidence (pLDDT). **(G)** Closeup of the predicted interaction between TPXL-1 and the nucleosome acidic patch, from a prediction of a *C. elegans* nucleosome octamer with the TPXL-1 CLD and a double-stranded DNA (Widom 601 sequence), colored by confidence (pLDDT). View is equivalent to **Fig. 3A** (inset). **(H)** AlphaFold 3 predicted aligned error (PAE) plot for a prediction of a complex of *C. elegans* H2A and H2B with the TPXL-1 CLD. **(I)** AlphaFold 3 predicted structure of a complex of *C. elegans* H2A and H2B with the TPXL-1 CLD, colored as in panel (*B*). **(J)** AlphaFold 3 predicted structure of a complex of *C. elegans* H2A and H2B with the TPXL-1 CLD, colored by confidence (pLDDT). **(K)** Closeup of the predicted interaction between TPXL-1 and the nucleosome acidic patch, from a prediction of *C. elegans* H2A and H2B with the TPXL-1 CLD, colored by confidence (pLDDT).

**Figure S3**

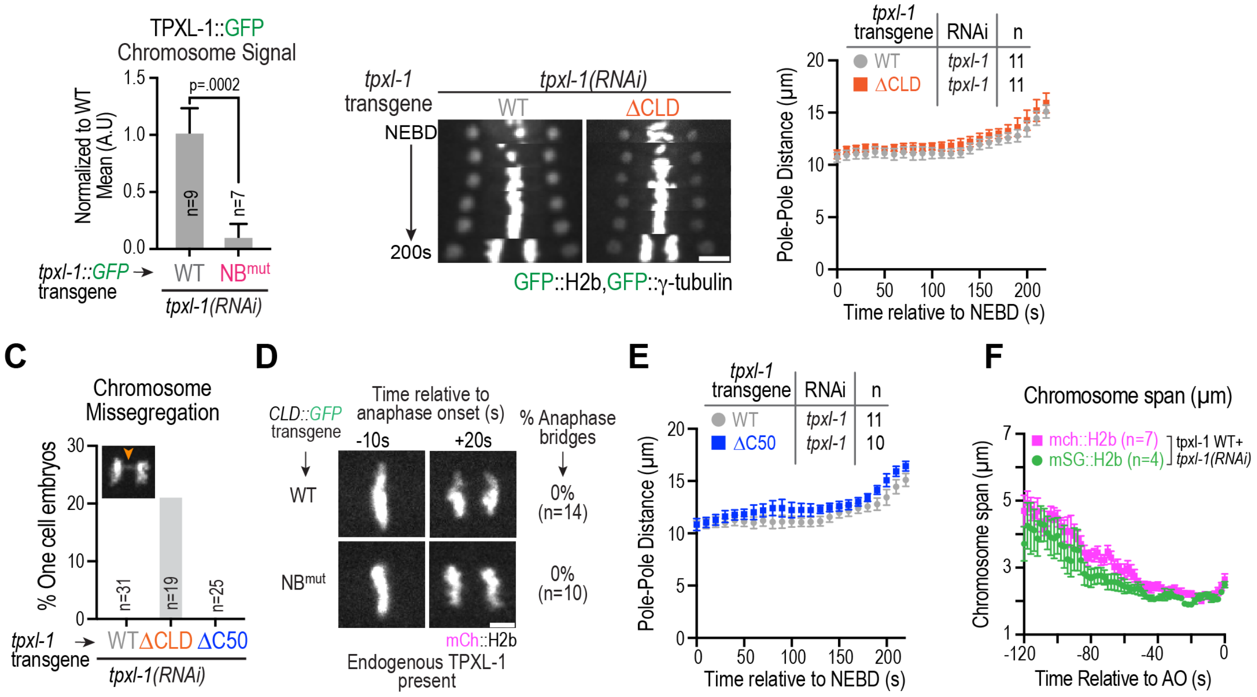


**Fig. S3. Additional localization and functional analysis of TPXL-1 variants. (A)** Quantification of localization of $NB^{mut}$ TPXL-1::GFP. *n* is the number of embryos imaged and analyzed. Error bars are the 95% CI. p-value (p=0.0002) is from a Mann-Whitney test. **(B)** (*left*) Kymograph of spindle region of embryos expressing GFP fusions to label chromosomes (H2b) and spindle poles (γ-tubulin). The indicated *tpxl-1* transgene insertions were present and endogenous TPXL-1 was depleted. Scale bar, 5 µm. (*right*) Graph of spindle pole separation over time for the indicated conditions. *n* is the number of one-cell embryos analyzed. Error bars are SD. **(C)** Graph plotting the percentage of one-cell embryos with chromatin bridges in anaphase for the indicated conditions. The embryos analyzed expressed GFP::H2b. *n* is the number of one-cell embryos analyzed. Note that the mCh::H2b and mSG::H2b analyzed for $NB^{mut}$ TPXL-1 (*Fig. 4C*) are more sensitive at detecting anaphase chromosome bridges (mCh::H2b, because of significantly lower autofluorescence background; mSG::H2b because it is significantly more photostable and brighter than GFP::H2b, which is also prone to expression silencing when expressed from the transgene integrated on Chr V), which may account for the lower frequency of visible anaphase chromosome bridges scored for ΔCLD relative to $NB^{mut}$ TPXL-1. **(D)** Analysis of chromosome segregation in the indicated conditions. Images are representative of time points immediately before (-10s) and after (+20s) anaphase onset in embryos expressing CLD:GFP transgenes with endogenous TPXL-1 present. Percent of embryos with anaphase bridges in each condition are indicated on the right. Scale bar, 2.5 µm. **(E)** Graph of spindle pole separation over time for the indicated conditions. *n* is the number of one-cell embryos analyzed. Error bars are SD. WT data are the same as in (*B*). **(F)** Chromosome span analysis separating out mCh::h2b from mSG::H2b embryos in the TPXL-1 WT + *tpxl-1(RNAi)* condition. Error bars are SEM. These data were pooled and plotted in *Fig. 5C*: WT + *tpxl-1(RNAi)*.

**Figure S4**

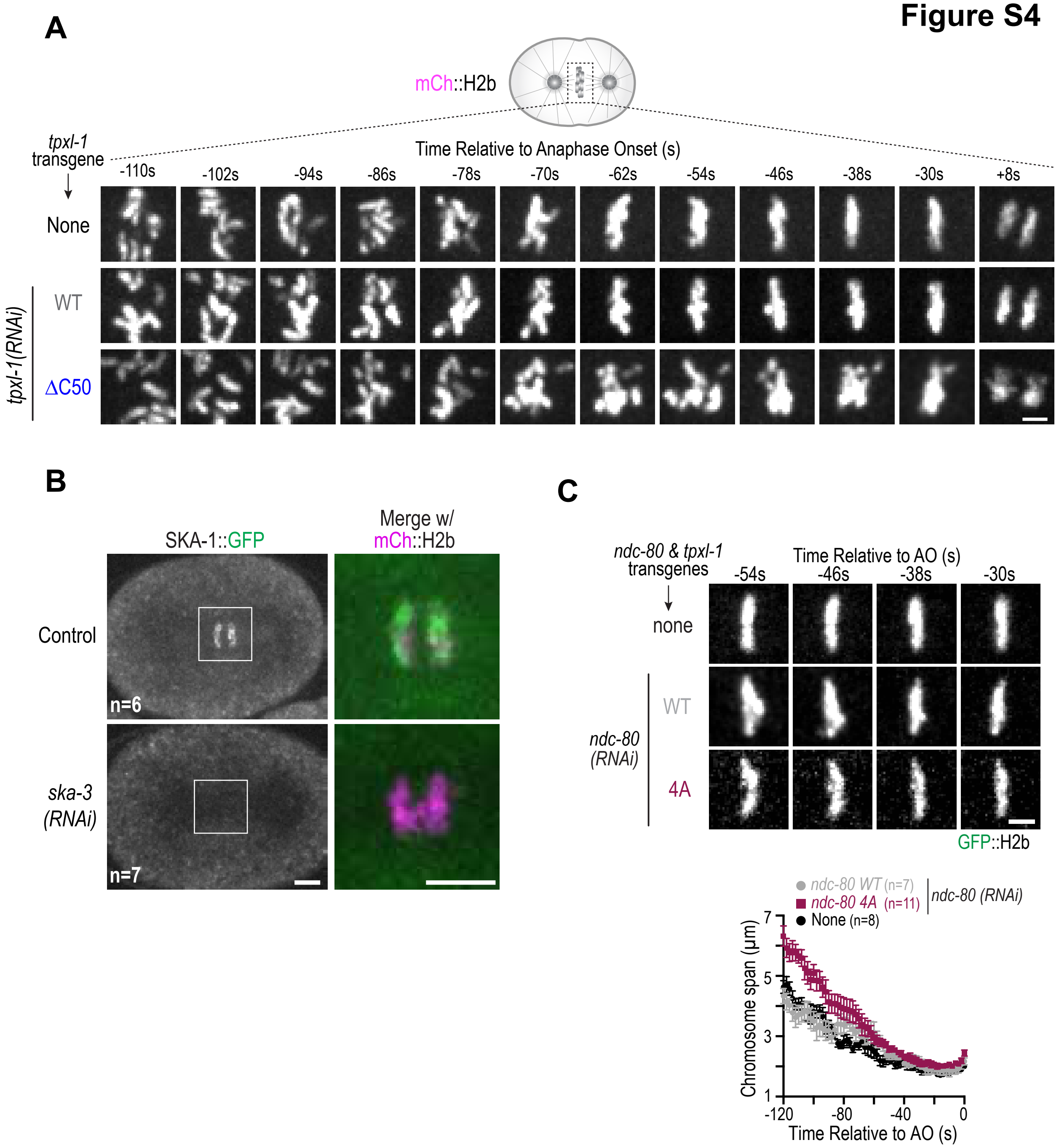


**Fig. S4. Additional analysis of chromosome dynamics and confirmation of SKA complex removal from kinetochores following SKA-3 depletion. (A)** Timelapse of chromosome dynamics in the indicated conditions. Scale bar, 2.5 µm. **(B)** Images of control and *ska-3(RNAi)* embryos with *in-situ* GFP-tagged SKA-1 that are expressing mCh::H2b. Chromosomal regions were boxed and magnified on the right. Scale bars, 5 µm for whole embryo and magnified panels. **(C)** Chromosome span analysis comparing WT to 4A NDC-80, following endogenous NDC-80 depletion. (*top*) Image panels from timelapse sequences for the indicated conditions. Scale bar, 2.5

μm. (*bottom*) Graph of chromosome span, analyzed as in *Fig. 5C*. Error bars are the SEM. Unlike WT NDC-80, NDC-80 4A mutant has chromosomes persisting near poles in early prometaphase which accounts for the initially larger chromosome span but they eventually congress to form a tight metaphase plate.

**Table S1. *C. elegans* strains**

| Strain | *Genotype* | Source |
|---|---|---|
| N2 | *Ancestral* | Caenorhabditis Genetics Center |
| OD1702 | *unc-119(ed3)III; ltSi560 [pPLG014; Pmex-5::GFP::his-11::tbb-2_3'UTR, tbg-1::gfp::tbb-2_3'UTR; cb-unc-119(+)]V* | Kim et al., 2015, PMID:25987605 (36) |
| OD5495 | *si24[pEZ145; pmex-5::TPXL-1 WT:: tbb-2; cb-unc-119(+)]II;unc-119(ed3)III; ltSi560 [oxTi365; oxTi365; pPLG014; Pmex-5::GFP::his-11::tbb-2_3'UTR, tbg-1::gfp::tbb-2_3'UTR; cb-unc-119(+)]V* | This Study |
| OD5971 | *si31[pEZ150; pmex-5:TPXL-1RE(A)FD::tbb-2; cb-unc-119(+)]II;unc-119(ed3) III; ltSi560 [oxTi365; oxTi365; pPLG014; Pmex-5::GFP::his-11::tbb-2_3'UTR, tbg-1::gfp::tbb-2_3'UTR; cb-unc-119(+)]V* | This Study |
| OD5146 | *tpxl-1(lt210[tpxl-1::lap::mNeonGreen]) I; ltIs37 [pAA64; pie-1/mCHERRY::his-58; unc-119 (+)] IV* | This Study |
| OD4932 | *unc-119(ed3) III? ; ltIs37 [pAA64; pie-1/mCHERRY::his-58; unc-119 (+)] IV; air-1(lt149[air-1::GFP])V* | This Study |
| OD5393 | *estSi121 [pEZ185; pmex-5::TPXL-1 WT::mNeonGreen:: tbb-2; cb-unc-119(+)]II; unc-119(ed3)III; knl-1( lt75[knl-1::mCherry]) III* | This Study |
| OD6379 | *si31[pEZ150; pmex-5:TPXL-1RE(A)FD::tbb-2; cb-unc-119(+)]II;unc-119(ed3)unc-119(ed3) III? ; ltIs37 [pAA64; pie-1/mCHERRY::his-58; unc-119 (+)] IV; air-1(lt149[air-1::GFP])V* | This Study |
| OD5922 | *si24[pEZ145; pmex-5::TPXL-1 WT:: tbb-2; cb-unc-119(+)]II;unc-119(ed3)III; air-1(lt149[air-1::GFP])V; ltIs37 [pAA64; pie-1/mCHERRY::his-58; unc-119 (+)] IV* | This Study |
| OD6380 | *estSi178 [pEZ231; pmex-5::TPXL-1FD::mNeonGreen:: tbb-2; cb-unc-119(+)]II; unc-119(ed3) III;ltIs37 [pAA64; pie-1/mCHERRY::his-58; unc-119 (+)] IV* | This Study |
| OD5153 | *estSi121 [pEZ185; pmex-5::TPXL-1WT::mNeonGreen:: tbb-2; cb-unc-119(+)] II ; ltIs37 [pAA64; pie-1/mCHERRY::his-58; unc-119 (+)] IV* | This Study |
| OD5970 | *ltSi1872[pJLM43; pmex-5:Tpxl-1 Reencoded::NeonGreen delta 205-257::tbb-2 3' UTR:cb-unc-119(+)II; ltIs37 [pAA64; pie-1/mCHERRY::his-58; unc-119 (+)] IV* | This Study |
| OD5923 | *ltSi1880[pJLM47; pmex-5:Tpxl-1 Reencoded::UT delta 205-257::tbb-2 3' UTR:cb-unc-119(+)]II; air-1(lt149[air-1::GFP])V; ltIs37 [pAA64; pie-1/mCHERRY::his-58; unc-119 (+)] IV* | This Study |
| OD6270 | *ltSi2033[pJLM57; pmex-5:Tpxl-1 Reencoded::205-257::GFP(pATC)::tbb-2 3' UTR:cb-unc-119(+)II; tIs37 [pAA64; pie-1/mCHERRY::his-58; unc-119 (+)] IV* | This Study |
| OD6382 | *ltSi2061[pJLM58; pmex-5:Tpxl-1 Reencoded::Randomized:205-257::GFP(pATC)::tbb-2 3' UTR:cb-unc-119(+)II; tIs37 [pAA64; pie-1/mCHERRY::his-58; unc-119 (+)] IV* | This Study |
| OD6383 | *ltSi2062[pJLM59; pmex-5:Tpxl-1 Reencoded::205-257 V223D, R226A::GFP(pATC)::tbb-2 3' UTR:cb-unc-119(+)II; tIs37 [pAA64; pie-1/mCHERRY::his-58; unc-119 (+)] IV* | This Study |
| OD6381 | *ltSi2023[pJLM52;Pmex-5::tpxl-1 WTRe-encoded::GFP(pATC)::tbb-2 3'UTR; cb-unc-119(+)]II; tIs37 [pAA64; pie-1/mCHERRY::his-58; unc-119 (+)] IV* | This Study |
| OD6384 | *ltSi2063[pJLM60; pmex-5:Tpxl-1 Reencoded V223D, R226A::GFP(pATC)::tbb-2 3' UTR:cb-unc-119(+)II; tIs37 [pAA64; pie-1/mCHERRY::his-58; unc-119 (+)] IV* | This Study |

| | | |
|---|---|---|
| OD6386 | *ltSi2065[pJLM61; pmex-5:Tpxl-1 Reencoded V223D, R226A::tbb-2 3' UTR:cb-unc-119(+)]II; ltIs37 [pAA64; pie-1/mCHERRY::his-58; unc-119 (+)] IV; air-1(lt149[air-1::GFP])V* | This Study |
| OD6387 | *si24[pEZ145; pmex-5::TPXL-1 WT:: tbb-2; cb-unc-119(+)]II;unc-119(ed3)III; ltSi2009[pOD5410/pAJS059; Pmex-5::TBG-1::Staygold::tbb-2 3'UTR_operonlinker_Staygold::His-11::tbb-2 3'UTR; cb-unc-119(+)] I ; unc-119(ed3) III* | This Study |
| OD6388 | *ltSi2065[pJLM61; pmex-5:Tpxl-1 Reencoded V223D, R226A::tbb-2 3' UTR:cb-unc-119(+)]II; ltSi2009[pOD5410/pAJS059; Pmex-5::TBG-1::Staygold::tbb-2 3'UTR_operonlinker_Staygold::His-11::tbb-2 3'UTR; cb-unc-119(+)] I ; unc-119(ed3) III* | This Study |
| OD5725 | *si24[pEZ145; pmex-5::TPXL-1 WT:: tbb-2; cb-unc-119(+)]II;unc-119(ed3)III ; (lt28[ska1::GFP]::loxp)I; ltIs37 [(pAA64) pie-1p::mCherry::his-58 + unc-119(+)] IV* | This Study |
| OD6389 | *ltSi2065[pJLM61; pmex-5:Tpxl-1 Reencoded V223D, R226A::tbb-2 3' UTR:cb-unc-119(+)]II; (lt28[ska1::GFP]::loxp)I; ltIs37 [(pAA64) pie-1p::mCherry::his-58 + unc-119(+)] IV* | This Study |
| OD5921 | *ltSi1741[pJLM2; pmex-5:Tpxl-1 Reencoded ΔC50::NeonGreen::tbb-2 3' UTR:cb-unc-119(+)]II;unc-119(ed3) III; ltIs37 [pAA64; pie-1/mCHERRY::his-58; unc-119 (+)] IV* | This Study |
| OD6067 | *ltSi1741[pJLM2; pmex-5:Tpxl-1 Reencoded ΔC50::tbb-2 3' UTR:cb-unc-119(+)]II; ltIs37 [pAA64; pie-1/mCHERRY::his-58; unc-119 (+)] IV; air-1(lt149[air-1::GFP])V* | This Study |
| OD2925 | *(lt28[ska1::GFP]::loxp)I; ltIs37 [(pAA64) pie-1p::mCherry::his-58 + unc-119(+)] IV* | Cheerambathur, et al., 2017 PMID: 28535376 (8) |
| OD5726 | *ltSi1745[pJLM20; pmex-5:Tpxl-1 Reencoded::ΔC50:tbb-2 3' UTR:cb-unc-119(+)]II ; (lt28[ska1::GFP]::loxp)I; ltIs37 [(pAA64) pie-1p::mCherry::his-58 + unc-119(+)] IV* | This Study |
| OD5725 | *si24[pEZ145; pmex-5::TPXL-1 WT Reencoded:: tbb-2; cb-unc-119(+)]II;unc-119(ed3)III ; (lt28[ska1::GFP]::loxp)I; ltIs37 [(pAA64) pie-1p::mCherry::his-58 + unc-119(+)] IV* | This Study |
| OD6000 | *ltSi2009[pOD5410/pAJS059; Pmex-5::TBG-1::Staygold::tbb-2 3'UTR_operonlinker_Staygold::His-11::tbb-2 3'UTR; cb-unc-119(+)] I ; unc-119(ed3) III* | This Study |
| OD6390 | *si31[pEZ150; pmex-5:TPXL-1RE(A)FD::tbb-2; cb-unc-119(+)]II;unc-119(ed3)unc-119(ed3) III?; (lt28[ska1::GFP]::loxp)I; ltIs37 [(pAA64) pie-1p::mCherry::his-58 + unc-119(+)] IV* | This Study |
| OD6391 | *ltSi2065[pJLM61; pmex-5:Tpxl-1 Reencoded V223D, R226A::tbb-2 3' UTR:cb-unc-119(+)]II; (lt28[ska1::GFP]::loxp)I; ltIs37 [(pAA64) pie-1p::mCherry::his-58 + unc-119(+)] IV* | This Study |
| OD6100 | *si24[pEZ145; pmex-5::TPXL-1 WT Reencoded:: tbb-2; cb-unc-119(+)]II; ltSi179[[pDC189;Pndc-80:NDC-80 reencoded; cb-unc-119(+)]I; unc-119(ed3)III; ltSi560 [oxTi365; oxTi365; pPLG014; Pmex-5::GFP::his-11::tbb-2_3'UTR, tbg-1::gfp::tbb-2_3'UTR; cb-unc-119(+)]V* | This Study |
| OD6098 | *ltSi1741[pJLM2; pmex-5:Tpxl-1 Reencoded ΔC50::tbb-2 3' UTR:cb-unc-119(+)]II; ltSi179[[pDC189;Pndc-80:NDC-80 reencoded; cb-unc-119(+)]I; unc-119(ed3)III; ltSi560 [oxTi365; oxTi365; pPLG014; Pmex-5::GFP::his-11::tbb-2_3'UTR, tbg-1::gfp::tbb-2_3'UTR; cb-unc-119(+)]V* | This Study |
| OD6101 | *si24[pEZ145; pmex-5::TPXL-1 WT Reencoded:: tbb-2; cb-unc-119(+)]II; ltSi502[pPLG005; Pndc-80::NDC-80 T8A,S18A,S44A,S51A re-encoded, cb-unc119(+)]I; unc-119(ed3)III; ltSi560 [oxTi365; oxTi365; pPLG014; Pmex-5::GFP::his-11::tbb-2_3'UTR, tbg-1::gfp::tbb-2_3'UTR; cb-unc-119(+)]V* | This Study |

| | | |
|---|---|---|
| OD6099 | *ltSi1741[pJLM2; pmex-5:Tpxl-1 Reencoded ΔC50::tbb-2 3' UTR:cb-unc-119(+)]II; ltSi502[pPLG005; Pndc-80::NDC-80 T8A,S18A,S44A,S51A re-encoded, cb-unc119(+)]I; unc-119(ed3)III; ltSi560 [oxTi365; oxTi365; pPLG014; Pmex-5::GFP::his-11::tbb-2_3'UTR, tbg-1::gfp::tbb-2_3'UTR; cb-unc-119(+)]V* | This Study |
| OD6392 | *si24[pEZ145; pmex-5::TPXL-1 WT Reencoded:: tbb-2; cb-unc-119(+)]II; ltSi179[[pDC189;Pndc-80:NDC-80 reencoded; cb-unc-119(+)]I; unc-119(ed3)III; (lt28[ska1::GFP]::loxp)I; ltIs37 [(pAA64) pie-1p::mCherry::his-58 + unc-119(+)] IV* | This Study |
| OD6393 | *ltSi1741[pJLM2; pmex-5:Tpxl-1 Reencoded ΔC50::tbb-2 3' UTR:cb-unc-119(+)]II; ltSi179[[pDC189;Pndc-80:NDC-80 reencoded; cb-unc-119(+)]I; unc-119(ed3)III; (lt28[ska1::GFP]::loxp)I; ltIs37 [(pAA64) pie-1p::mCherry::his-58 + unc-119(+)] IV* | This Study |
| OD6394 | *ltSi1741[pJLM2; pmex-5:Tpxl-1 Reencoded ΔC50::tbb-2 3' UTR:cb-unc-119(+)]II; ltSi502[pPLG005; Pndc-80::NDC-80 T8A,S18A,S44A,S51A re-encoded, cb-unc119(+)]I; unc-119(ed3)III; (lt28[ska1::GFP]::loxp)I; ltIs37 [(pAA64) pie-1p::mCherry::his-58 + unc-119(+)] IV* | This Study |
| OD5730 | *ltSi1880[pJLM47; pmex-5:Tpxl-1 Reencoded::UT Δ205-257::tbb-2 3' UTR:cb-unc-119(+)]II ; unc-119(ed3)III; ltSi560 [oxTi365; oxTi365; pPLG014; Pmex-5::GFP::his-11::tbb-2_3'UTR, tbg-1::gfp::tbb-2_3'UTR; cb-unc-119(+)]V* | This Study |
| OD6395 | *ltSi179[[pDC189;Pndc-80:NDC-80 reencoded; cb-unc-119(+)]I; unc-119(ed3)III; ltSi560 [oxTi365; oxTi365; pPLG014; Pmex-5::GFP::his-11::tbb-2_3'UTR, tbg-1::gfp::tbb-2_3'UTR; cb-unc-119(+)]V* | This Study |
| OD6396 | *ltSi502[pPLG005; Pndc-80::NDC-80 T8A,S18A,S44A,S51A re-encoded, cb-unc119(+)]I; ltSi560 [oxTi365; oxTi365; pPLG014; Pmex-5::GFP::his-11::tbb-2_3'UTR, tbg-1::gfp::tbb-2_3'UTR; cb-unc-119(+)]V* | This Study |

**Table S2. Primers for dsRNA synthesis**

| Gene No. | Name | Oligonucleotide (5'-3'), #1 | Oligonucleotide (5'-3') #2 | Template |
|---|---|---|---|---|
| Y39G10AR .12 | *tpxl-1* | AATTAACCCTCACTAAAGGTG TACACATATGATGGCACAGG | TAATACGACTCACTATAGGA CGTCGGTGAGCAAATTGAC | N2 cDNA |
| W01B6.9 | *ndc-80* | AATTAACCCTCACTAAAGGGA TGACAAGTACATTCAGAGATT ATACAAATGATC | TAATACGACTCACTATAGGG TGGTTCAAGATTCATTTGAA TATTAAGTCCACTG | N2 cDNA |
| F58A4.3 | *hcp-3* | AATTAACCCTCACTAAAGGAC ACCCCAATTATTGAGGAAATC GCCGAGC | TAATACGACTCACTATAGGC GAAGGCAGAGACGTCTGTA TAACTGAATATCC | N2 gDNA |
| Y39G10AR .13 | *icp-1* | AATTAACCCTCACTAAAGGCG TCAACACCTGGACGTATG | TAATACGACTCACTATAGGG ATAGGTACGTGGCGGAGTC | yk329a |
| F35G12.8 | *smc-4* | AATTAACCCTCACTAAAGGTG CATCTTCTTCTTTCCCTACA | TAATACGACTCACTATAGGC TCCAAAACAAGCCGAACTT | N2 gDNA |
| F54E7.8 | *ska-3* | AATTAACCCTCACTAAAGGTT ATGGATAATAGAAAGTCAACG | TAATACGACTCACTATAGGA TGGAATCGTTTATTGATCGG AT | N2 gDNA |

## SUPPLEMENTAL MOVIE LEGENDS

### Movie S1.

Time-lapse movies of the nuclear envelope breakdown through anaphase onset interval in one-cell embryos co-expressing GFP::TBG-1 (γ-tubulin) and GFP::H2b (histone H2b). The two conditions shown are transgene-expressed TPXL-1 WT transgene (*left*) and TPXL-1 $AurAInt^{mut}$ (*right*) following endogenous TPXL-1 depletion (*tpxl-1 RNAi*). Images were acquired at 10s intervals. Playback is 10 frames per second (=100x real-time). Related to **Fig. 1D.**

### Movie S2.

Movies of one-cell embryos expressing mCherry::H2b. Both movies begin at -40s relative to anaphase onset. (*left*) Embryos expressing TPXL-1 WT and (*right*) TPXL-1 $NB^{mut}$ following endogenous TPXL-1 depletion (*tpxl-1 RNAi*). Images were acquired at 10s intervals. Playback is 5 frames per second (=50x real-time). Related to **Fig. 4C**.

### Movie S3.

High-speed (*2s interval*) imaging of one-cell embryos expressing mCherry::H2b for the indicated conditions. Movie begins at -120s relative to anaphase onset. Playback is 10 frames per second (=20x real-time). Related to **Fig. 5C** and **Fig. 6A**.

## REFERENCES


1. J. K. Monda, I. M. Cheeseman, The kinetochore-microtubule interface at a glance. *J. Cell Sci.* **131**, jcs214577 (2018).

2. A. Musacchio, A. Desai, A molecular view of kinetochore assembly and function. *Biology* **6**, 5 (2017).

3. H. Hochegger, N. Hegarat, J. B. Pereira-Leal, Aurora at the pole and equator: Overlapping functions of Aurora kinases in the mitotic spindle. *Open Biol.* **3**, 120185 (2013).

4. S. Li, T. Kasciukovic, T. U. Tanaka, Kinetochore-microtubule error correction for biorientation: Lessons from yeast. *Biochem. Soc. Trans.* **52**, 29–39 (2024).

5. M. A. Lampson, I. M. Cheeseman, Sensing centromere tension: Aurora B and the regulation of kinetochore function. *Trends Cell Biol.* **21**, 133–140 (2011).

6. M. Carmena, M. Wheelock, H. Funabiki, W. C. Earnshaw, The chromosomal passenger complex (CPC): From easy rider to the godfather of mitosis. *Nat. Rev. Mol. Cell Biol.* **13**, 789–803 (2012).

7. H. Abdelkabir, S. W. Arachchige, S. P. Wheatley, Survivin and Aurora kinase A control cell fate decisions during mitosis. *Mol. Oncol.* **20**, 727–752 (2025).

8. D. K. Cheerambathur, B. Prevo, N. Hattersley, L. Lewellyn, K. D. Corbett, K. Oegema, A. Desai, Dephosphorylation of the Ndc80 tail stabilizes kinetochore-microtubule attachments via the ska complex. *Dev. Cell* **41**, 424–437.e4 (2017).

9. L. Chmatal, K. Yang, R. M. Schultz, M. A. Lampson, Spatial regulation of kinetochore microtubule attachments by destabilization at spindle poles in meiosis I. *Curr. Biol.* **25**, 1835–1841 (2015).

10. K. F. DeLuca, A. Meppelink, A. J. Broad, J. E. Mick, O. B. Peersen, S. Pektas, S. M. A. Lens, J. G. DeLuca, Aurora A kinase phosphorylates Hec1 to regulate metaphase kinetochore-microtubule dynamics. *J. Cell Biol.* **217**, 163–177 (2018).

11. G. Eot-Houllier, L. Magnaghi-Jaulin, G. Fulcrand, F. X. Moyroud, S. Monier, C. Jaulin, Aurora A-dependent CENP-A phosphorylation at inner centromeres protects bioriented chromosomes against cohesion fatigue. *Nat. Commun.* **9**, 1888 (2018).

12. N. Ozlu, M. Srayko, K. Kinoshita, B. Habermann, E. T. O'Toole, T. Muller-Reichert, N. Schmalz, A. Desai, A. A. Hyman, An essential function of the *C. elegans* ortholog of TPX2 is to localize activated Aurora A kinase to mitotic spindles. *Dev. Cell* **9**, 237–248 (2005).

13. T. Sobajima, K. M. Kowalczyk, S. Skylakakis, D. Hayward, L. J. Fulcher, C. Neary, C. Batley, S. Kurlekar, E. Roberts, U. Gruneberg, F. A. Barr, PP6 regulation of Aurora A-TPX2 limits NDC80 phosphorylation and mitotic spindle size. *J. Cell Biol.* **222**, e202205117 (2023).

14. A. A. Ye, J. Deretic, C. M. Hoel, A. W. Hinman, D. Cimini, J. P. Welburn, T. J. Maresca, Aurora A kinase contributes to a pole-based error correction pathway. *Curr. Biol.* **25**, 1842–1851 (2015).

15. N. Tavernier, F. Sicheri, L. Pintard, Aurora A kinase activation: Different means to different ends. *J. Cell Biol.* **220**, e202106128 (2021).

16. M. Toya, M. Terasawa, K. Nagata, Y. Iida, A. Sugimoto, A kinase-independent role for Aurora A in the assembly of mitotic spindle microtubules in *Caenorhabditis elegans* embryos. *Nat. Cell Biol.* **13**, 708–714 (2011).

17. S. Mangal, J. Sacher, T. Kim, D. S. Osorio, F. Motegi, A. X. Carvalho, K. Oegema, E. Zanin, TPXL-1 activates Aurora A to clear contractile ring components from the polar cortex during cytokinesis. *J. Cell Biol.* **217**, 837–848 (2018).

18. S. Redemann, J. Baumgart, N. Lindow, M. Shelley, E. Nazockdast, A. Kratz, S. Prohaska, J. Brugues, S. Furthauer, T. Muller-Reichert, *C. elegans* chromosomes connect to centrosomes by anchoring into the spindle network. *Nat. Commun.* **8**, 15288 (2017).

19. J. Espeut, D. K. Cheerambathur, L. Krenning, K. Oegema, A. Desai, Microtubule binding by KNL-1 contributes to spindle checkpoint silencing at the kinetochore. *J. Cell Biol.* **196**, 469–482 (2012).

20. J. Abramson, J. Adler, J. Dunger, R. Evans, T. Green, A. Pritzel, O. Ronneberger, L. Willmore, A. J. Ballard, J. Bambrick, S. W. Bodenstein, D. A. Evans, C. C. Hung, M. O'Neill, D. Reiman, K. Tunyasuvunakool, Z. Wu, A. Žemgulytė, E. Arvaniti, C. Beattie, O. Bertolli, A. Bridgland, A. Cherepanov, M. Congreve, A. I. Cowen-Rivers, A. Cowie, M. Figurnov, F. B. Fuchs, H. Gladman, R. Jain, Y. A. Khan, C. M. R. Low, K. Perlin, A. Potapenko, P. Savy, S. Singh, A. Stecula, A. Thillaisundaram, C. Tong, S. Yakneen, E. D. Zhong, M. Zielinski, A. Žídek, V. Bapst, P. Kohli, M. Jaderberg, D. Hassabis, J. M. Jumper, Accurate structure prediction of biomolecular interactions with AlphaFold 3. *Nature* **630**, 493–500 (2024).

21. Y. Mito, A. Sugimoto, M. Yamamoto, Distinct developmental function of two *Caenorhabditis elegans* homologs of the cohesin subunit Scc1/Rad21. *Mol. Biol. Cell* **14**, 2399–2409 (2003).

22. L. L. Moore, G. Stanvitch, M. B. Roth, D. Rosen, HCP-4/CENP-C promotes the prophase timing of centromere resolution by enabling the centromere association of HCP-6 in *Caenorhabditis elegans*. *Mol. Cell. Biol.* **25**, 2583–2592 (2005).

23. R. K. McGinty, S. Tan, Principles of nucleosome recognition by chromatin factors and enzymes. *Curr. Opin. Struct. Biol.* **71**, 16–26 (2021).

24. K. I. Lange, A. Suleman, M. Srayko, Kinetochore recruitment of the spindle and kinetochore-associated (Ska) complex is regulated by centrosomal PP2A in *Caenorhabditis elegans*. *Genetics* **212**, 509–522 (2019).

25. M. Rosas-Salvans, C. J. Rux, M. Das, S. Dumont, SKAP binding to microtubules reduces friction at the kinetochore-microtubule interface and increases attachment stability under force. *Curr. Biol.* **35**, 1805–1815.e4 (2025).

26. T. Kim, P. Lara-Gonzalez, B. Prevo, F. Meitinger, D. K. Cheerambathur, K. Oegema, A. Desai, Kinetochores accelerate or delay APC/C activation by directing Cdc20 to opposing fates. *Genes Dev.* **31**, 1089–1094 (2017).

27. A. W. Bird, A. A. Hyman, Building a spindle of the correct length in human cells requires the interaction between TPX2 and Aurora A. *J. Cell Biol.* **182**, 289–300 (2008).

28. C. Frøkjær-Jensen, M. W. Davis, M. Sarov, J. Taylor, S. Flibotte, M. LaBella, A. Pozniakovsky, D. G. Moerman, E. M. Jorgensen, Random and targeted transgene insertion in *Caenorhabditis elegans* using a modified Mos1 transposon. *Nat. Methods* **11**, 529–534 (2014).

29. M. D. Aljohani, S. El Mouridi, M. Priyadarshini, A. M. Vargas-Velazquez, C. Frøkjær-Jensen, Engineering rules that minimize germline silencing of transgenes in simple extrachromosomal arrays in *C. elegans*. *Nat. Commun.* **11**, 6300 (2020).

30. M. Hirano, R. Ando, S. Shimozono, M. Sugiyama, N. Takeda, H. Kurokawa, R. Deguchi, K. Endo, K. Haga, R. Takai-Todaka, S. Inaura, Y. Matsumura, H. Hama, Y. Okada, T. Fujiwara, T. Morimoto, K. Katayama, A. Miyawaki, A highly photostable and bright green fluorescent protein. *Nat. Biotechnol.* **40**, 1132–1142 (2022).

31. E. Ivorra-Molla, D. Akhuli, M. B. L. McAndrew, W. Scott, L. Kumar, S. Palani, M. Mishima, A. Crow, M. K. Balasubramanian, A monomeric StayGold fluorescent protein. *Nat. Biotechnol.* **42**, 1368–1371 (2024).

32. A. Paix, A. Folkmann, D. Rasoloson, G. Seydoux, High efficiency, homology-directed genome editing in *Caenorhabditis elegans* using CRISPR-Cas9 ribonucleoprotein complexes. *Genetics* **201**, 47–54 (2015).

33. K. Luger, T. J. Rechsteiner, T. J. Richmond, Expression and purification of recombinant histones and nucleosome reconstitution. *Methods Mol. Biol.* **119**, 1–16 (1999).

34. C. Elfmann, J. Stulke, PAE viewer: A webserver for the interactive visualization of the predicted aligned error for multimer structure predictions and crosslinks. *Nucleic Acids Res.* **51**, W404–W410 (2023).

35. W. L. DeLano, PyMOL: An open-source molecular graphics tool. *CCP4 Newsletter Protein Crystallogr.* **40**, 44–53 (2002).

36. T. Kim, M. W. Moyle, P. Lara-Gonzalez, C. De Groot, K. Oegema, A. Desai, Kinetochore-localized BUB-1/BUB-3 complex promotes anaphase onset in *C. elegans*. *J. Cell Biol.* **4**, 507–517 (2015).
